# Task-based and Magnified Mirror Therapy for Unilateral Spatial Neglect among post-stroke subjects: Study protocol for a randomized controlled trial

**DOI:** 10.1371/journal.pone.0296276

**Published:** 2024-01-24

**Authors:** Kamal Narayan Arya, Shanta Pandian, Divya Pandey, G. G. Agarwal, Neera Chaudhary

**Affiliations:** 1 Department of Occupational Therapy, Pandit Deendayal Upadhyaya National Institute for Persons with Physical Disabilities, New Delhi, India; 2 Department of Statistics, Lucknow University, Lucknow, Uttar Pradesh, India; 3 Department of Neurology, Govind Ballabh Pant Institute of Postgraduate Medical Education and Research, New Delhi, India; Universite Catholique de Louvain, BELGIUM

## Abstract

**Background:**

Unilateral spatial neglect (USN) is a commonly occurring neurocognitive disability after a stroke. The neglect may affect the motor recovery of the upper and lower limbs and functional performances. Mirror therapy, a simple and economical approach has the potential to reduce the USN and related impairments.

**Aim:**

The primary objective of this study is to determine the effectiveness of task-based and magnified mirror therapy on the USN and on the motor recovery of the post-stroke subjects. The secondary objective is to investigate the effectiveness of the intervention on the function and disability of the subjects.

**Methods:**

In this randomized controlled, assessor-blinded trial, 86 post-stroke subjects will be recruited from the neuro-rehabilitation laboratory of a rehabilitation institute, located in northern India. The participants,aged20 to 80 years, with 1 to 36 months of stroke onset, hemiparesis, and the USN, will be considered eligible for the study. In addition to the conventional rehabilitation, the experimental group(n = 43) will receive 40 sessions (8 weeks) of **T**ask-based and **MAG**nified **M**irror Therapy for **U**nilateral **S**patial **N**eglect (T-MAGUSN). The control group (n = 43) will undergo a dose-matched conventional program only. The participants will be assessed at baseline, post-intervention and 4-week follow-up using primary (Line Bisection Test, Letter Cancellation Test, and Fugl-Myer Assessment) and secondary (Catherine Bergego Scale, Berg Balance Scale, Functional Ambulation Classification, Modified Rankin Scale) outcome measures.

**Discussion:**

This proposed study will lead to the development of a novel rehabilitation protocol for the management of USN, aiming to enhance motor and functional recovery. The investigation will consider both the upper and lower limbs for the intervention, reducing the impact of cognitive disability in stroke.

**Trial registration:**

Clinical Trial Registry of India (CTRI) as *CTRI/2023/05/053184* (www.ctri.nic.in/Clinicaltrials/pmaindet2.php?trialid=74659).

## Introduction

Unilateral spatial neglect (USN), commonly occurring after a stroke is considered to be a neuropsychological or cognitive disorder [1, 2]. The terms unilateral neglect, spatial neglect, spatial inattention, visuospatial neglect, and hemineglect are used interchangeably. Post-stroke subjects with USN are unable to recognize, react, and regulate the information present in the space of the body side contralateral to the brain lesion. USN may also be considered a deficit in awareness of stimuli such as visual or auditory in the contralesional space. In comparison to USN, hemianopia is a visual field deficit on the contralesional side considering the midline orientation [3]. The subject may experience USN in various forms; for instance, the inability to perform functions by the paretic limb (impaired voluntary motor control of the limb contralateral to the brain lesion) [4] or any other body part in space of the paretic side, in spite of having adequate motor ability. USN may be categorized into 10 different subtypes, ranging from motor to sensory and personal to extrapersonal space [5]. Spatially, USN may be observed in the nearest region of the body space to the farthest area in relation to the paretic side. About 70% of the subjects with USN exhibit neglect in the reaching space that may have a strong functional implication [6].

The reported prevalence of USN among individuals who had a stroke varies among the studies, due to the type of tests used and the chronicity of the subjects [7]. Though more common in those with right brain hemisphere damage, neglect may be observed among individuals with left brain involvement. The occurrence varies from44% to85% and 12.5% to 20% respectively, among the left and right paretic subjects [8]. The impact of USN may reduce as the subject reaches the chronic stage [7].

USN affects upper limb usage in daily functions as well as motor recovery of the limb. The neglect has been found to be associated with the upper limb paresis, dexterity, and goal-directed movements and activities of daily living [9, 10]. Longitudinally, the neglect negatively influences the recovery pattern, especially by restraining the recovery potential during the initial window period [11].

USN may be considered an important determinant for trunk control [12]. Further, the USN may be influenced by body position. Postures such as standing demanding lower limb muscles may exhibit a lessened impact of USN as compared to the sitting position [13]. However, due to altered midline orientation individuals with USN exhibit an abnormal crossed-leg sign (overlapping of the right leg on the left leg to provide perceptual information) during sitting position [14]. Due to the neglect, the post-stroke subjects experience altered deviation in the mediolateral stability during standing or walking, leading to poor balance [15]. The subjects with USN are unable to modulate a variety of proprioceptive inputs that may be required for appropriate motor control [16]. The neglect may also lead an awkward and asymmetrical gait, altered goal-directed locomotion and reduced recovery potential for functional ambulation such as stair climbing [17–19]. Thus, USN not only affects upper limb function but also balance and performance allied to the trunk and lower limb. USN is considered to be a common cognitive deficit that substantially contributes to post-stroke disability [20]. USN not only increases the functional disability of the individuals who had a stroke but also adds to the burden on the family or caretaker [21].

Rehabilitation intervention for USN ranges from robotics to virtual reality to prism adaptation training. The robotic training may enhance midline orientation altered due to neglect; however, the training induces no significant effect on functional performance [22]. The virtual reality intervention has potential for the USN, yet good quality randomized trials are lacking [23]. The prism adaptation may reduce the USN; however, the favourable effects are short lasting [24]. Furthermore, the focus of most of these therapies is on the paretic upper limb.

Mirror therapy, a simple and economical approach has potential for lessening the USN as well as related functions; a structured and specific therapy protocol still needs to be developed [25, 26]. Only one systematic review [26] of the five studies (238 subjects) determined the effect of mirror therapy in the USN. The review indicates evidence for mirror therapy in reducing the USN and enhancing activities of daily living though high quality evidence is warranted. Further, none of the included studies evaluated the effect of mirror therapy on motor recovery. Some of these studies used multiple tasks to impart mirror therapy. The task- or activity-based interventions are found to be useful for the USN rehabilitation [27]. Further, task-based mirror therapy also exhibited favourable motor recovery in stroke [28, 29]. In addition to this, the magnified vision during post-stroke motor therapy improves tactile discrimination required during a motor task [30]. In a single-group study, post-stroke subjects could better perform tasks such as reaching and grasping in the condition of magnified vision [31].

Thus, USN is a common disabling manifestation further affecting the motor recovery of post-stroke subjects. The **T**ask-based and **MAG**nified **M**irror Therapy for **U**nilateral **S**patial **N**eglect (T-MAGUSN) based on tasks and magnification of vision as well as for both paretic upper and lower limbs has potential for managing the USN. The parallel neurobiological mechanism for the paretic limb and USN requires the rehabilitation of multi-fold action [32]. Mirror therapy can lead to visuo-tactile integration by stimulating numerous brain areas such as the bilateral premotor, primary motor cortex, primary somatosensory cortex, and cerebellum to reorganize the functions of damaged brain [33, 34]. The use of tasks in mirror therapy also increases the activity of mirror neurons, which are considered to play a crucial role in the regime. Mirror therapy also has potential to augment impaired body representation in stroke [35]. Further, magnifying the image of the hand or fingers enlarges the cortical representation of the body part increasing the underlying neuromechanism of mirror therapy [30, 31]. Additionally, the use of mirror therapy in the parasagittal plane would provide visual feedback for the affected limb and neglected space [36].

The T-MAGUSN investigation will have implication for real-world life and the trial will be conducted in a clinical setting [37]. The primary objective of this study is to determine the effectiveness of T-MAGUSN on the reduction of USN and the improvement of the motor recovery of the subjects. The secondary objective is to investigate the effectiveness of the intervention on the function and disability of the subjects. It is hypothesized that, compared with conventional rehabilitation, the T-MAGUSN would substantially diminish the impact of USN on the entire body and augment the motor recovery of the paretic limbs, reducing the disability among post-stroke subjects.

## Methods

### Design

**Fig 1:**
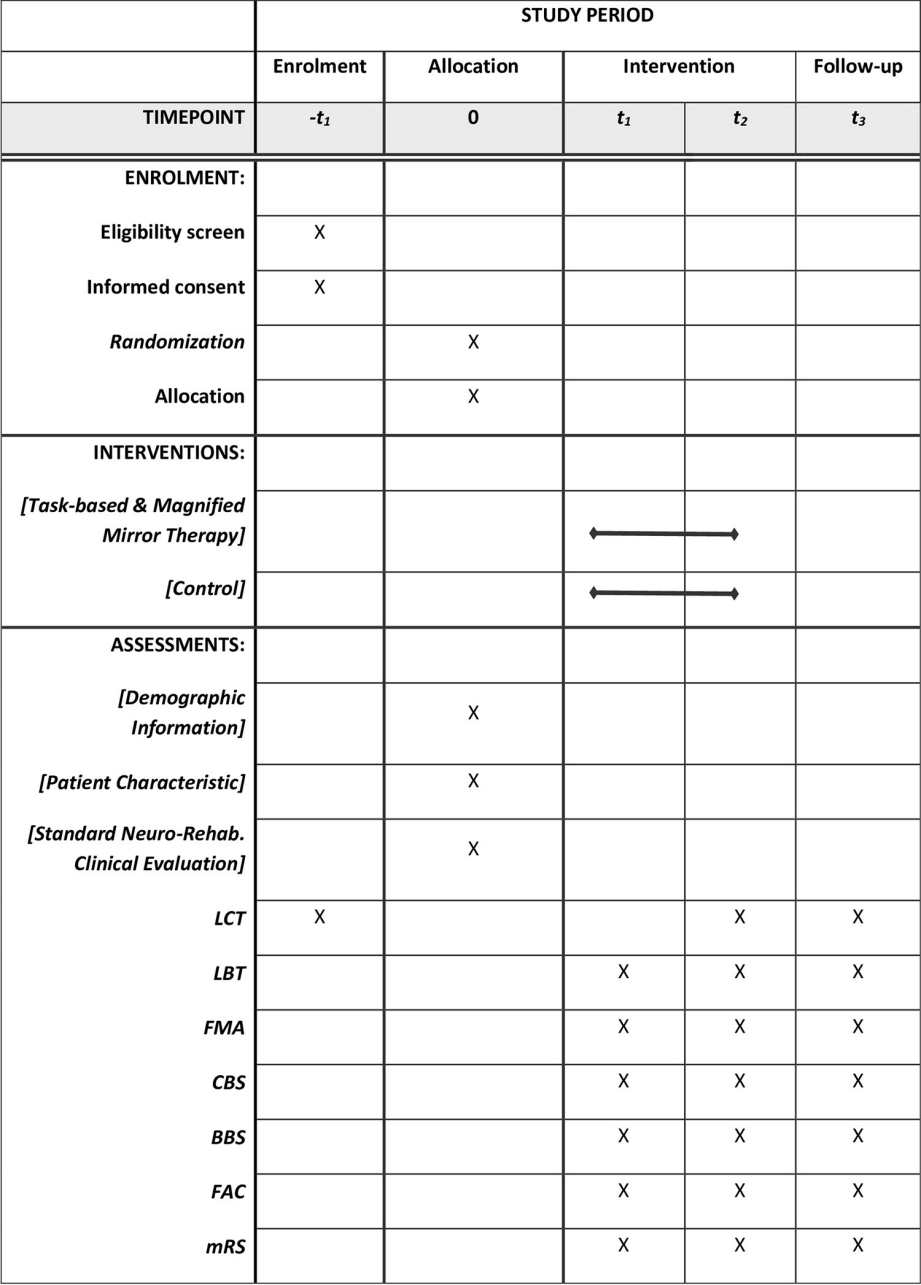
The timeline for schedule of enrolment, intervention, and assessment as per SPIRIT guidelines. t1 baseline assessment, t2 assessment after 8 weeks of intervention, t3 assessment after four-week follow-up (12 weeks after start of intervention). LCT = Letter Cancellation Task, LBT = Line Bisection Test, FMA = Fugl-Meyer assessment, CBS = Catherine Bergego Scale, BBS = Berg Balance Scale, FAC = Functional Ambulation Category, mRS = modified Rankin scale.

**Fig 2:**
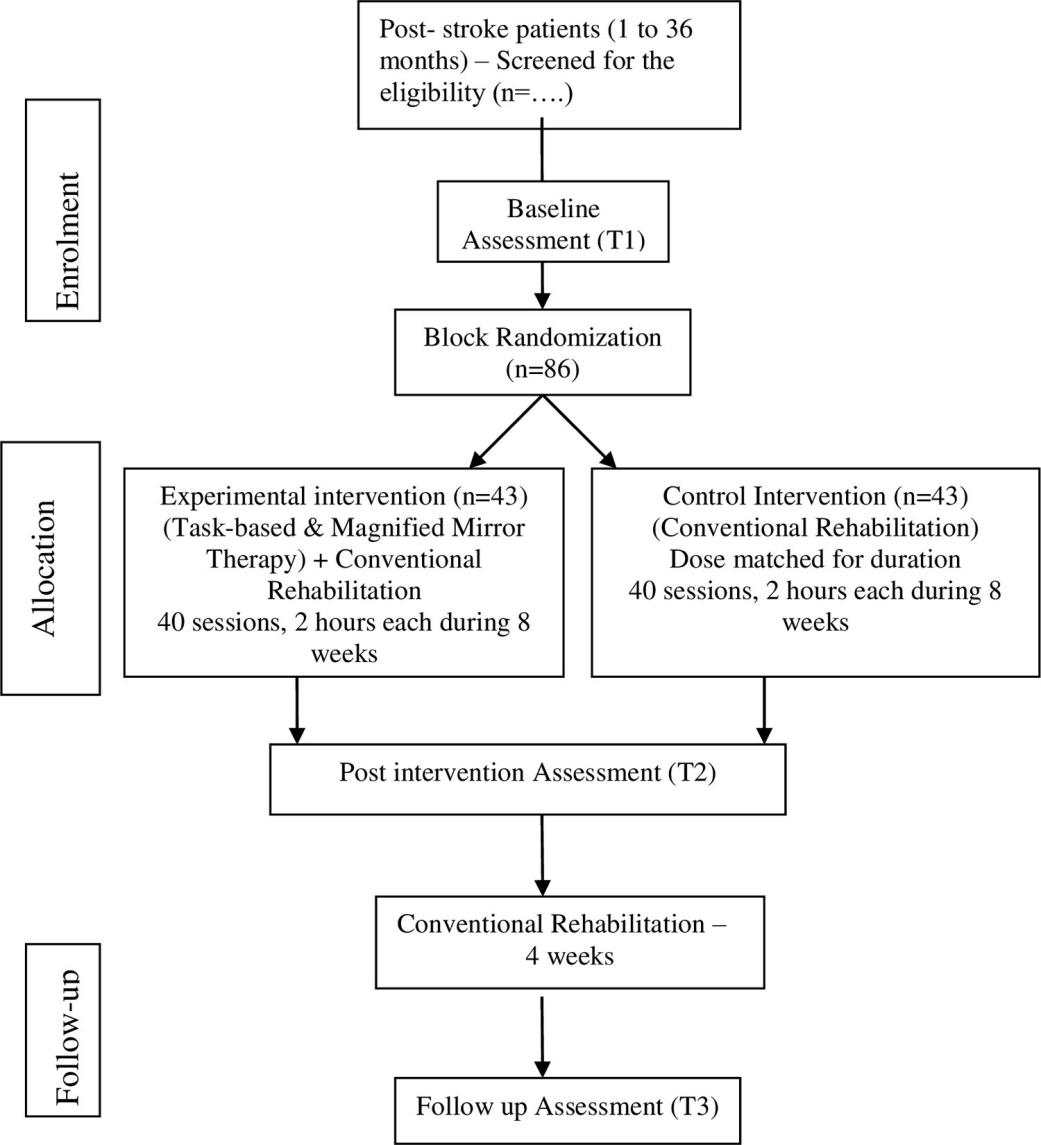
Flow of the study process.

The study will be a randomized controlled, assessor blinded, and superiority trial. The timeline for the schedule of enrolment, intervention, and assessment as per SPIRIT guidelines [38] is provided in Fig 1. The flow of the study process is shown in Fig 2.

### Setting

Neuro-rehab laboratory, department of occupational therapy of a national-level institute for persons with physical disabilities situated in New Delhi, India.

### Eligibility criteria

The subjects will be recruited if they exhibit the following criteria: 1) 20 to 80 years of age, 2)post-stroke duration of 1 to 36 months, 3) hemiparesis of either the right or left side of the body(as assessed by Fugl-Meyer assessment [39] upper extremity subsection: 0 to 66), 4) USN (asymmetry between the Letter cancellation task omissions in the left and right visual fields of at least 2 letters) [32], 5) Normal visual abilities (6/6 visual acuity with or without glasses and corrected hyperopia, if present), and 6) Muscle ≤ grade 2 on the modified Ashworth scale. However, the subjects will be excluded if they have any of the following: 1) receptive communication or other language disorder interfering with the assessment and treatment process (National Institute of Health Stroke Scale; item 9 ≥ 2), 2) severe cognitive impairment (Mini-Mental State Examination≤ 17) [40], 3) homonymous hemianopia (confrontation test) [41], 4) contractures and deformities of the hand, 5) complex regional pain syndrome (clinically diagnosed), [42] and 6) severe depression (Beck depression inventory > 30) [43, 44].

### Sample size

The power calculation was done using the values for USN assessment from a study on mirror therapy for lessening the USN in stroke [25]. In a previous mirror therapy study for the USN in stroke, only one of the primary outcome measures (the Line Bisection Test) was found to be used. Thus, the values of mean difference = 8.6 and SD = 10.6 (as measured by the Line Bisection Test) between the experimental and control groups, with beta = 0.1and α = 0.05, were used for the sample size estimation. The calculation indicated that at least 37 subjects in each group would be required to determine the expected change due to the experimental intervention. Anticipating a drop out of 15%, 43 subjects in each group (a total 86 participants) will be enrolled in this study. The potential participants will be recruited from the outpatient unit of the study site as well as referral will be received from the neurology department of a super speciality state government hospital to ensure a sufficient sample size.

### Ethical approval

The present study has been ethically endorsed by the institutional ethics committee of the Pandit Deendayal Upadhyaya National Institute for Persons with Physical Disabilities, New Delhi, India (IEC9/2020/RP3; dated 04^th^ March 2020 and 24^th^ May 2023) [45]. All the study participants will be provided with a patient information sheet to understand the purpose and other aspects of the investigation. Accordingly, they will be signing the informed written consent form.

### Group allocation, concealment, and blinding

The recruited participants will be allocated into either the experimental group or the control group in block of 10 (with the last block of 6). An assessor, who will be unacquainted with the allotted group of study participants, will apply the outcome measures at baseline, post-intervention, and follow-up. A computer professional, not associated with the study will apply the randomization process using the SPSS Version 23 software. The treatment protocols will be assigned in the ratio of 1:1. The assignment will be consecutively placed in sealed, opaque envelopes. Considering the content of the experimental protocol, the blinding of the clinician or subject will not be possible.

### Recruitment status of the trial

This single-centre trial is open to recruitment from June 2023 and is expected to be completed for a calculated sample size by the end of 2025.

### Experimental intervention

The present study will comprise an experimental program, **T**ask-based and **MAG**nified **M**irror Therapy for **U**nilateral **S**patial **N**eglect (T-MAGUSN), primarily based on mirror therapy using tasks and magnification of vision. The TIDieR (Template for Intervention Description and Replication) checklist [46] has been applied to ensure the completeness of intervention.

**Fig 3:**
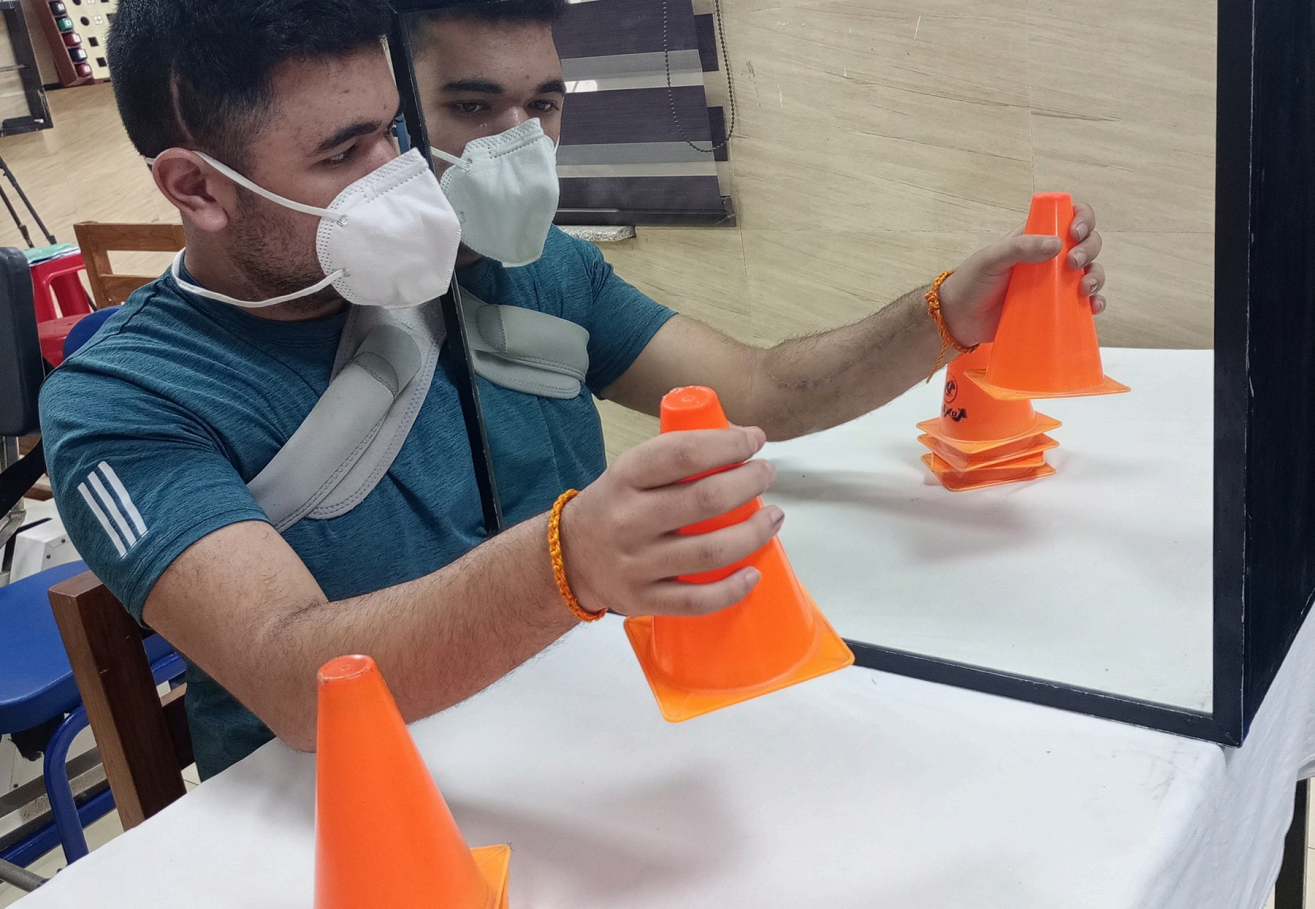
Cone stacking task by the non-paretic upper limb.

**Fig 4:**
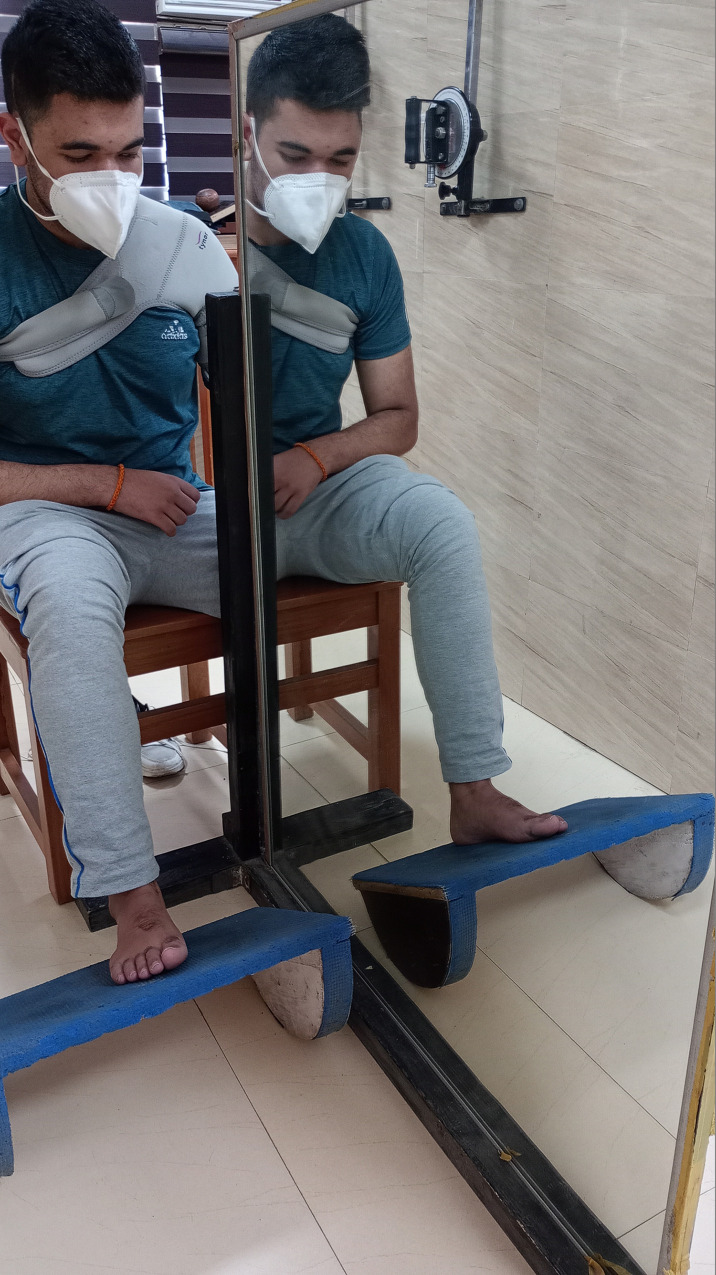
Rocker board activity by the non-paretic lower limb.

**Fig 5:**
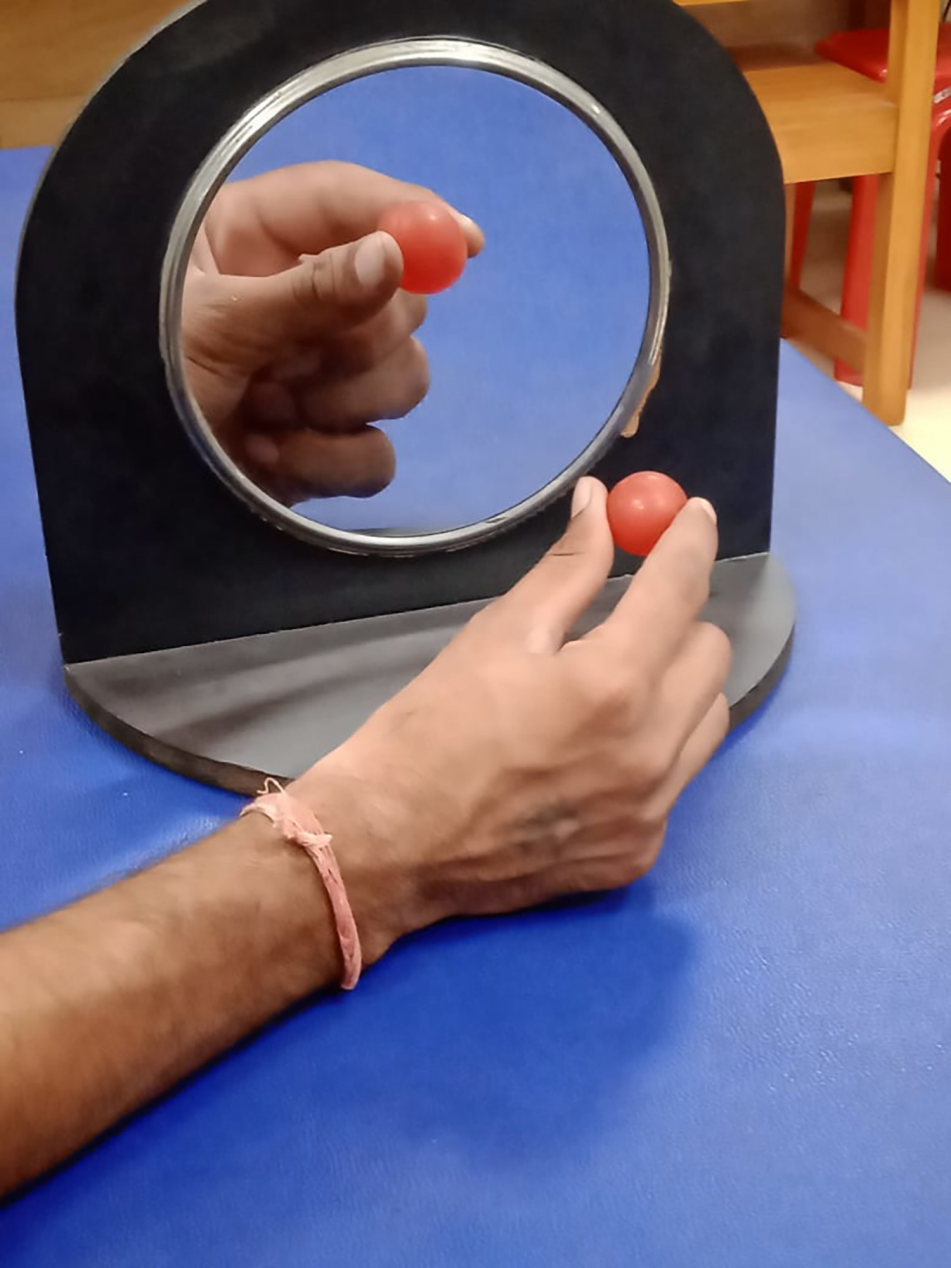
Magnified prehensile activity by the non-paretic hand.

**Fig 6:**
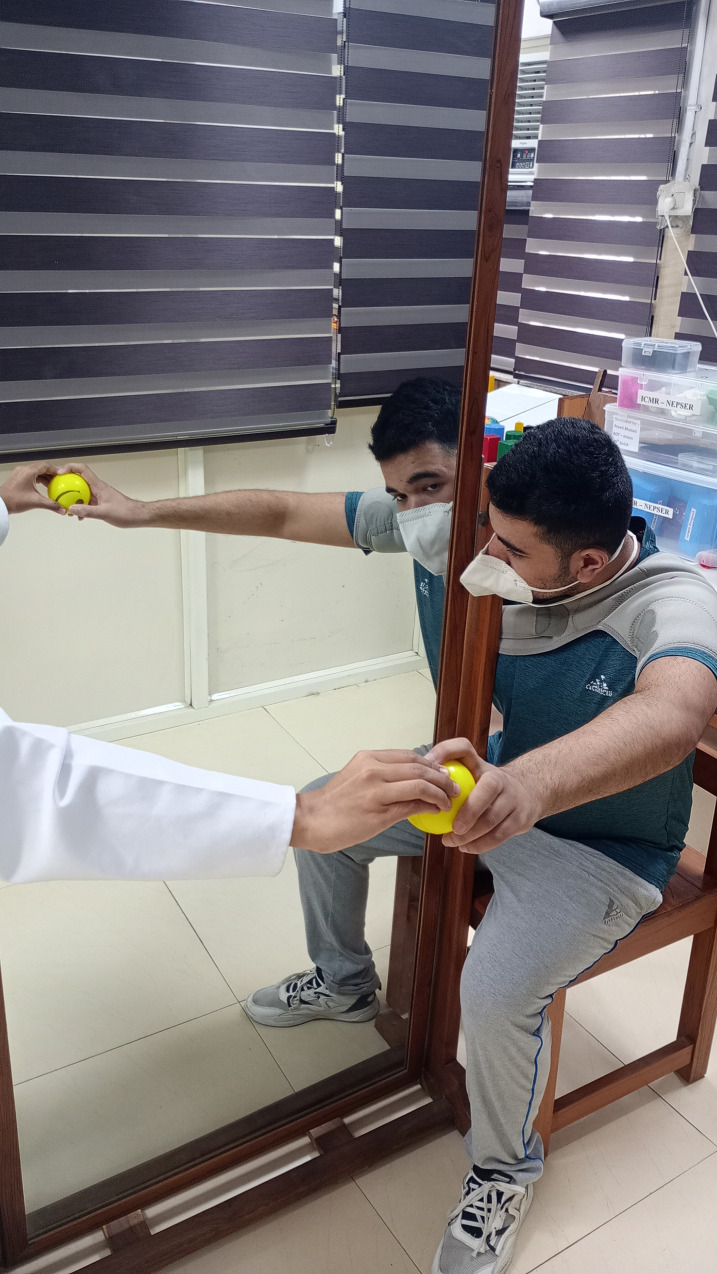
Visual feedback of the paretic upper limb performance in the parasagittal plane.

The duration of the program will be 1 hr, 40 sessions across 8 weeks. In addition to the T-MAGUSN, conventional motor rehabilitation of 1 hr will also be imparted [47]. The regime will be provided in a distraction-free environment in the neuro-rehabilitation lab of the study site. The T-MAGUSN intervention will comprise the following 4 components(Figs 3 to 6 showing a left hemiparetic subject with unilateral spatial neglect performing the mirror therapy; *The individual pictured in Figs 3 to 6 has provided written informed consent (as outlined in PLOS consent form) to publish their image alongside the manuscript)*:

1. Task-based upper limb mirror therapy(20 minutes x 40 sessions)
  The protocol guidelines will be followed as per our previous work [28] on upper limb motor recovery using task-based mirror therapy. However, necessary modifications in terms of USN as an objective of the intervention will be carried out. A wooden mirror box or frame with dimensions of 30x18x40 inches will be used. Various objects, such as pegs, blocks, clay, ball, U-clips, soft balls, and stacking cones will be used to provide the mirror therapy. The textured objects [48] will also be provided to provide multi-sensory awareness. The participant will be made to sit close to a table on which the mirror box or frame will be placed vertically at the level of the sternum. The less-affected upper limb will be placed in front of the mirror to visualize the image while the more-affected limb will be placed inside the mirror box. The subject could perceive the image of the less-affected limb as the affected limb in the mirror. By observing the image of the less-affected upper extremity as a projection over the affected limb, an illusion will be created. The tasks will be provided with an objective to perceive the body part, movement, and object in the ipsilesional space as the illusion of the contralesional space. The objects will be reached for, grasped, lifted, placed, and manipulated in different spatial planes (forward/backward, superior/inferior, medially/laterally, diagonally). The therapist will instruct for the direction and may locate the object in the desired plane by standing in front of the patient. The movements of all the joints (shoulder, elbow, forearm, wrist, hand and fingers) will be used in view of the associated motor impairment. The number of tasks, joints, repetition, speed, time and resistance will be considered as parameters for grading the intervention. The movements will be imparted unimanually when the weakened limb has no voluntary or abnormal synergistic movements, while the movements will be carried out bimanually by the subjects if the impaired limb has out-of-synergy control.
2. Task-based lower limb mirror therapy(20 minutes x 40 sessions)
  For the lower limb mirror therapy, the protocol will be followed as per our previous study [29]. A short-sitting mirror frame having dimensions of 24 x 72 inches will be used with a provision for the mirror to be tilted in the sagittal plane to view the mirror at an acute angle, between 75 and 85 degrees. The arrangement will provide the maximum possible mirror perception of the moving limb with negligible sight of the other body part. Another frame measuring 36 x 48 inches will be used for the long-sitting activity. The subject will receive task-based movements such as rocker or wobble board (ankle), foot roller (knee and ankle), picking up sticks and curling cloth by the toes, and shifting a pillow or towel by the less-affected side. The activities will be graded as described previously for the upper limb program.
3. Magnified mirror therapy (10 minutes x 40 sessions)
  To provide the intervention, a magnified (2–3 times) mirror will be used. The task-based therapy, guidelines as described earlier for the upper limb intervention, will be followed. The hand and finger manipulation using various meaningful objects will be used by the less-paretic hand to provide the illusion for the paretic hand. The subject will be instructed to minutely visualize the magnified body parts and objects and then manipulate the objects. The movements will be performed indifferent spatial planes. The number of tasks, repetition, speed, time and resistance will be considered as parameters for grading the intervention. The magnified mirror therapy will not be provided for the upper arm and lower limb.
4. Task-based parasagital plane visual feedback mirror therapy(10 minutes x 40 sessions)

A mirror frame will be positioned vertically on the less-affected side with a therapist standing behind it. The mirror frame will be held parallel to the sagittal plane so that its right edge will be close to the patient’s paretic shoulder. The subject will be asked to turn his head and eyes to look into the centre of the mirror so that he can clearly view the mirror reflection of the people or objects that are on the paretic side. A therapist standing on the patient’s paretic side will hold an object such as a block or cone and move towards the subject so that it is well within the reach of the subject’s non-paretic side but entirely within the neglected (paretic) visual field about 8 inches below and to the paretic side nose. The therapist will then enquire about the object and ask the patient to reach for the item. Similarly, the objects will be positioned for reach and manipulation in different locations in the neglected field. The number of objects, repetition, speed, time and resistance will be considered as parameters for grading the intervention. This form of feedback based mirror therapy will be provided to the subjects when they will demonstrate out-of-synergy movements on the paretic side.

Conventional rehabilitation [47, 49] will comprise motor therapy for the paretic upper limbs using enabling and functional activities such as reaching, grasping and manipulating objects, sandings, and prehensile activities. Weight bearing on the paretic upper limb and bilateral upper limb movements will also be provided Shoulder care and the prescription of shoulder support for prevention and management of shoulder subluxation will also be considered. The prescription of ankle foot orthosis and quadripod will be mainly followed for the paretic lower limb. Sit-to-stand, weight shift and functional walking will also be imparted.

### Control intervention

In standard clinical practice at the study site, the USN is being managed jointly with the motor paresis. Motor and functional activities are provided primarily using the principles of mid-line crossing and visual feedback. Thus, in addition to the conventional motor rehabilitation as mentioned for the experimental group, the control subjects will be provided following intervention [47, 49] for the USN rehabilitation for a dose match (2 hr x 40 sessions, 8 weeks) program–

1. A mirror frame will be placed in front of the patient while he will be performing activities of daily living, ambulation and motor activities (unilateral and bilateral) in order to draw attention to the neglected side.
2. Mid-line crossing activities: unilateral and bilateral.
3. Scanning, puzzles, trail making, and drawing activities.

The experimental and control interventions will be delivered by the investigators; K.N.A. and S.P., respectively. Both of them have more than 20 years of experience in the field of stroke rehabilitation. In order to have good adherence to the intervention, the subjects will be provided a travel allowance during the intervention period, as approved by the funding agency.

### Post trial care

After the completion of the 40 sessions of interventions, both groups will receive conventional rehabilitation for another 4 weeks.

### Primary outcome measures

#### Unilateral spatial neglect

*a*. *Line Bisection Test*. Line Bisection Test (LBT) consists of three 20-cm horizontal black lines drawn on anA4 sheet of paper. The first line is drawn on the right side of the page, the second line is in the centre, and the third line is marked on the left side. The subject will be seated on a chair and the paper will be securely clipped to a board placed exactly in front of the individual on a table. The subject will be instructed to bisect each line while avoiding any movement of his head. The error of the bisection will be measured in cm. The left side inaccuracy will be scored with negative numbers while the right side inaccuracy will be awarded positive numbers. The cut-off score for unilateral visual neglect will be considered an error of more than 1.4 cm left or right. It is a reliable and valid measure of USN and a commonly used measure in stroke studies [25, 50].

*b*. *Letter Cancellation Test*. The Letter Cancellation Test (LCT) also assesses USN. The test comprises the cancellation of O’s on an A4 sheet securely placed as described earlier for the LBT. The sheet has 40 O’s (20 in the contralesional and 20 in the ipsilesional visual field) and 425 distractor letters. An asymmetry between the omissions in the contralesional and ipsilesional visual fields of at least 2 is considered to be the USN. LCT exhibits adequate psychometric properties and is usually applied in stroke investigations [32, 51, 52].

#### Motor recovery

*Fugl-Meyer assessment*. The Fugl-Meyer assessment [39] (FMA) is a gold standard performance-based measure of motor recovery. The motor section of FMA is arranged hierarchically and evaluates reflexive, synergistic, mixed synergistic, out-of-synergy movements and coordination. The motor section is further divided into two subsections: the upper extremities (upper arm and wrist and hand) and the lower extremities. The upper extremity (FMA-UE) subsection will be used to assess upper extremity recovery in the present study. It is scored out of 66, with sub-score of 36 for the upper arm (FMA-UA) and 30 for the wrist and hand (FMA-WH). Most of the items are scored on a 3-point ordinal scale, from 0 (no function) to 2 (full function). The lower-extremity subsection (FMA-LE) has a total of 17 items, which are scored similarly to FMA-UE. The FMA has been found to have excellent reliability and validity to quantify motor recovery among post-stroke hemiparetic subjects [53–55].

### Secondary outcome measures

#### Catherine Bergego Scale

The Catherine Bergego Scale (CBS) is used to measure functional neglect.CBS measures neglect through observation of activities such as eating, dressing, and walking. The CBS has items that assess personal, peripersonal, and extrapersonal neglect. The 10-item scale is scored using a 4-point scale (0 to 3); a higher score indicate the greater severity of neglect. It is a valid, reliable, and sensitive tool to measure the USN in stroke [56, 57].

#### Berg Balance Scale

The Berg Balance Scale (BBS) is used to assess balance ability among post-stroke subjects [58]. BBS is a 14-item measure and comprises items such as sit-to-stand, reaching, and standing on one foot. The items are scored on a 5-point scale (0 to 4); a higher score indicates good balance. The maximum score is 56, with < 40 and < 20 being considered medium and high fall risk, respectively for an individual. BBS demonstrates adequate psychometric properties to assess balance among stroke subjects [59].

#### Functional Ambulation Category

Functional Ambulation Category (FAC) measures the walking ability of post-stroke subjects. It is a simple method to quantify independence in ambulation. The scale categorise ambulation into 6 levels, ranging from 0 (unable to walk) to 5 (independent walking). FAC has exhibited acceptable reliability and validity [60, 61].

#### Modified Rankin scale

The modified Rankin scale (mRS) is a gold standard measure to categorize disability level in stroke [62]. It is a most common measure in stroke related studies and is considered to be an end-point outcome for the trials. mRS scores range from 0 (no symptoms at all) to 5 (severe disability). The scale exhibits excellent reliability and validity [63].

### Data collection

The data will be collected using the assessment proforma which comprises items and questions related to demographic aspects of the subjects such as age, gender, type of stroke, duration of stroke, area of involvement, and functional performance. Additionally, the clinical evaluation of a standard neuro-rehabilitation condition will also be covered. All the standardized outcome measures in the form of data collection tools will also be used.

### Data analysis

The collected data will be entered in MS-Excel spread sheet which will be further imported into IBM SPSS version 23.0 for analysis. The clinical and demographic characteristics and baseline features of the recruited subjects will be estimated in the form of mean (SD) or median (IQR) or n (%). Either Mann-Whitney U (U) or independent t (t) or chi-square (χ^2^)] tests will be executed to analyze the difference in characteristics between the experimental and control groups. An intention-to-treat analysis method will be followed by carrying forward the last observation. A repeated-measures 2-way ANOVA (continuous data; within factor, time; between factor, group) will be applied to study the difference for the post-intervention and follow-up scores of primary (LBT, LCT, and FMA) and secondary outcome measures (CBS, and BBS) between the experimental and control groups. The FAC and mRS will be reported in terms of n (%) for each level or category, which will be analysed using χ^2^test. In addition to the significance level of *p* < .05, 95% CI of the mean difference for the measures will also be reported. The primary analysis will also be expanded considering the age group, gender, side of involvement, and chronicity of stroke as covariates. In view of heterogeneity among the stroke subjects, logarithmic transformation of the data will be done for analysis of the variables that will not be normally distributed.

### Data management

Data from clinical assessments will be recorded in paper booklets for each patient and converted with double data entry into a PC data file stored on a secured server at the site of study. A copy of the data will also be stored on a secured pen drive on a weekly basis. A data code sheet will be developed, giving a unique code to each piece of information and a numerical scoring system for each of them. All the data will be securely stored for a period of 5 years.

### Data monitoring

The proposed study will not have a data monitoring committee in view of no or negligible adverse events from the experimental intervention. Further, there will not be any interim analysis or stopping guidelines for the investigation.

### Adverse events

Based on a previous stroke study [25], it is understood that there is no or very low risk of adverse events in this proposed investigation. If any adverse event is observed during the protocol, the same will be reported to the institutional ethics committee and described in the dissemination of the work. The experimental intervention may be temporarily or permanently discontinued for the concerned participant considering the adverse event or as decided by the committee.

### Protocol amendments

Any modifications in the proposed protocol related to the study objectives and/or methods that may influence the study outcome and/or benefit or harm to the participants will be informed to the institutional ethics committee and the funding agency. The necessary approval in this regard will also be taken from both the bodies.

### Confidentiality

All data collected during this study will be kept confidential other than for the investigators and research staff at the study site. In order to protect the privacy of the participants, a unique identification code will be provided to them. All assessment forms, score sheets, medical, and other reports will be coded and kept securely to maintain confidentiality.

### Dissemination

The findings of the study will be disseminated in the form of an oral or poster presentation at a national or international conference related to rehabilitation, neurology or occupational therapy to be held in India. Further, the outcome of the investigation will be published in a peer reviewed indexed journal of repute related to stroke rehabilitation. The CONSORT (Consolidated Standards of Reporting Trials) guidelines for the Non-pharmacologic treatments will be followed for the reporting of the study findings [64].

## Discussion

This proposed study will lead to the development of a novel rehabilitation protocol for the management of USN in stroke. The investigation will consider both the upper and lower limbs for the intervention. The regime primarily based on mirror therapy will comprise tasks and magnification of vision. Both the task and magnification are novel concepts infused into the mirror therapy. The task-based mirror therapy and magnified vision have already been used in stroke rehabilitation [28, 30]. The underlying mechanisms of these techniques are complementary to each other and are assumed to expand the recovery process among stroke survivors with USN [33]. The protocol may reduce the impact of cognitive disability among post-stroke survivors by reducing USN and enhancing the motor and functional recoveries. The recovery may further lessen the impact of overall stroke disability and enhance the quality of life of the post-stroke survivors. Stroke being a condition with varied features affecting the recovery; it will not be possible to recruit an appropriate number of homogenous subjects considering the type of stroke, lesioned cortex area, and motor recovery status. The results from this study will be unable to determine the level of illusion out of mirror therapy that would reduce the USN. The subtype of USN deficit and related measure is already a challenge [5]. The present investigation will use three measures of USN, including a tool covering the impact on functions. Further, in view of drop outs, structured rehabilitation during follow-up and other patient related issues such as transportation, the follow-up period of 4 weeks is planned.

The findings of the present study, if favourable, may be investigated for other neurocognitive deficits among post-stroke patients. The protocol may be refined considering the various types of USN. Further, the USN protocol of mirror therapy may be integrated with an already established mirror-based motor regime.

## Supporting information

S1 Checklist SPIRIT 2013 checklist: Recommended items to address in a clinical trial protocol and related documents*. (DOC)

S2 Checklist The TIDieR (Template for Intervention Description and Replication) checklist*: Information to include when describing an intervention and the location of the information. (DOCX)

S1 File (DOCX)

## Abbreviations

USN: Unilateral spatial neglect
T-MAGUSN: Task-based andMAGnifiedMirror Therapy for Unilateral Spatial Neglect
LBT: Line Bisection Test
LCT: Letter Cancellation Test
FMA: Fugl-Meyer Assessment
CBS: Catherine Bergego Scale
BBS: Berg Balance Scale
FAC: Functional Ambulation Category
mRS: modified Rankin Scale
