# Supplementary material for: Task-based and Magnified Mirror Therapy for Unilateral Spatial Neglect among post-stroke subjects: Study protocol for a randomized controlled trial
Source: PLoS One. 2024 Jan 24;19(1):e0296276. doi: 10.1371/journal.pone.0296276 (PMC10807845; doi:10.1371/journal.pone.0296276)
Supplement: S1 Checklist — (DOC) [file pone.0296276.s001.doc]

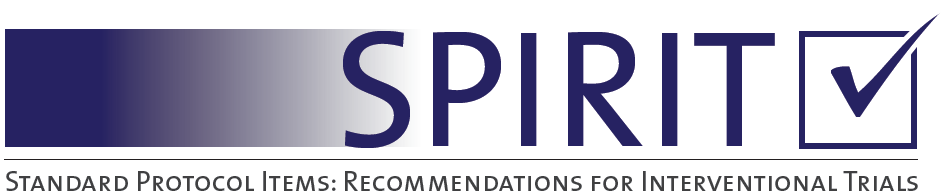


SPIRIT 2013 Checklist: Recommended items to address in a clinical trial protocol and related documents*

| Section/item | ItemNo | Page No. |
| --- | --- | --- |
| **Administrative information** | | |
| Title | 1 | Page 1 |
| Trial registration | 2a | Page 4 |
|  | 2b | Page 10 |
| Protocol version | 3 | -- |
| Funding | 4 | As per the journal protocol the information is provided in the online submission system only. |
| Roles and responsibilities | 5a | Page 1 & 22 |
|  | 5b | Page 1 |
|  | 5c | Page 1 |
|  | 5d | -- |
| Introduction |  |  |
| Background and rationale | 6a | Page 5 to 8 |
|  | 6b | Page 14 |
| Objectives | 7 | Page 8 |
| Trial design | 8 | Page 9 |
| Methods: Participants, interventions, and outcomes | | |
| Study setting | 9 | Page 9 |
| Eligibility criteria | 10 | Page 9 |
| Interventions | 11a | Page 10 to 15 |
|  | 11b | Page 19 |
|  | 11c | Page 14 |
|  | 11d | Page 14 &15 |
| Outcomes | 12 | Page 15 to 17 |
| Participant timeline | 13 | Page 17, Figure 2 |
| Sample size | 14 | Page 9 &10 |
| Recruitment | 15 | Page 10 |
| **Methods: Assignment of interventions (for controlled trials)** | | |
| Allocation: |  |  |
| Sequence generation | 16a | Page 10 |
| Allocation concealment mechanism | 16b | Page 10 |
| Implementation | 16c | Page 10 |
| Blinding (masking) | 17a | Page 10 |
|  | 17b | Page 10 |
| **Methods: Data collection, management, and analysis** | | |
| Data collection methods | 18a | Page 18 |
|  | 18b | Page 18 |
| Data management | 19 | Page 18 & 19 |
| Statistical methods | 20a | Page 18 |
|  | 20b | Page 18 |
|  | 20c | Page 18 |
| **Methods: Monitoring** | | |
| Data monitoring | 21a | Page 19 |
|  | 21b | Page 19 |
| Harms | 22 | Page 19 |
| Auditing | 23 | Page 19 |
| Ethics and dissemination | | |
| Research ethics approval | 24 | Page 10 |
| Protocol amendments | 25 | Page 19 |
| Consent or assent | 26a | Page 10 |
|  | 26b | -- |
| Confidentiality | 27 | Page 19 & 20 |
| Declaration of interests | 28 | Page 2 |
| Access to data | 29 | Page 1 |
| Ancillary and post-trial care | 30 | Page 14 & 15 |
| Dissemination policy | 31a | Page 20 |
|  | 31b | -- |
|  | 31c | -- |
| Appendices |  |  |
| Informed consent materials | 32 | -- |
| Biological specimens | 33 | -- |

*It is strongly recommended that this checklist be read in conjunction with the SPIRIT 2013 Explanation & Elaboration for important clarification on the items. Amendments to the protocol should be tracked and dated. The SPIRIT checklist is copyrighted by the SPIRIT Group under the Creative Commons “[Attribution-NonCommercial-NoDerivs 3.0 Unported](http://www.creativecommons.org/licenses/by-nc-nd/3.0/)” license.
