# Supplementary material for: Task-based and Magnified Mirror Therapy for Unilateral Spatial Neglect among post-stroke subjects: Study protocol for a randomized controlled trial
Source: PLoS One. 2024 Jan 24;19(1):e0296276. doi: 10.1371/journal.pone.0296276 (PMC10807845; doi:10.1371/journal.pone.0296276)
Supplement: S1 File — (DOCX) [file pone.0296276.s003.docx]

**RESEARCH PROPOSAL**

**(Submitted to Institutional Ethics Committee, Pandit Deendayal Upadhyaya National Institute for Persons with Physical Disabilities, New Delhi, India)**

**________________________________________________________**

### Principal Investigator: Dr. Kamal Narayan Arya, Lecturer (Occupational Therapy), PDUNIPPD

1. **Title:** Task-based and Magnified Mirror Therapy for Unilateral Spatial Neglect among Post-Stroke Subjects: A Randomized Controlled Trial
2. **Summary:**

Background: Up to 90% of the post-stroke survivors may experience the Unilateral Spatial Neglect (USN). USN is a deficit of attention and is described as the inability to report, respond, or orient to sensory or visual stimuli when it appears on the side opposite to the lesion. USN affects motor recovery, functional performance, and quality of life.

Novelty: The proposed study would be the first investigation to utilize the task-based and magnified mirror therapy for both upper and lower limbs and visual feedback by positioning mirror in parasagital position.

Objective: The primary objective is to determine the effectiveness of Task-based and magnified mirror therapy on Unilateral Spatial Neglect (USN) as well as motor recovery among post-stroke hemiparetic subjects.

Methods:

Design: Randomized controlled, single blinded trial

Setting**:** Neuro-Rehab Laboratory

Inclusion criteria: Age: 20 to 80 years, ischemic or hemorrhagic stroke, 1 to 36 months post-stroke, hemiparesis (right or left), USN (an asymmetry between the Letter cancellation task).

Exclusion Criteria: Severe other cognitive impairment, homonymous hemianopia, contractures and deformities of hand / finger, Severe depression.

Sample size: 86

Intervention: Experimental group will receive 40 sessions, 5/week, during 2 months, 1 hour experimental intervention in addition to 1 hour conventional rehabilitation. Experimental intervention will comprise of task-based and magnified mirror therapy for both upper and lower limbs in addition to the visual feedback. Control group will receive dose matched standard rehabilitation only.

Outcome measure: Letter cancellation task, Line bisection test, Fugl-meyer assessment, Catherine Bergego scale, Functional ambulation category, Berg balance scale, Modified Rankin scale.

Data analysis: A repeated-measures 2-way ANOVA will be used to study difference for the post intervention (8-week) and follow-up (4-week) scores between the groups.

Expected outcomes: The proposed study will lead to development of a novel rehabilitation protocol for the management of USN in stroke. The protocol may be favorable in reducing the impact of cognitive disability among post-stroke survivors by reducing USN and enhancing the motor and functional recovery.

1. **Background:**

According to the World Health Organization, more than 15 million people experience stroke worldwide each year. Of these, 5 million die and another 5 million gets permanently disabled.[^1^](#_ENREF_1) 70% of strokes occur in low- or middle-income countries (LMICs) such as India. Consequently, the disability burden in LMICs is greater than that of the high-income countries. Further, it has been projected to cause 23 million first-ever strokes, 77 million stroke survivors, and 61 million DALYs by 2030 in LMICs. Thus, the condition has considerable socioeconomic impact on the patients, family members and health care providers, posing a major public health challenge.[^2^](#_ENREF_2) Presently, the stroke incidence in India is much higher than the western developed countries.[^3^](#_ENREF_3)^,^ [^4^](#_ENREF_4) Stroke incidence ranged from 100 to 150/100,000 every year whereas the prevalence is up to 550/100,000 in various states of India.[^5-8^](#_ENREF_5) Due to upward trend in incidence during the last few decades, the disease has now been considered as an epidemic in India.[^7^](#_ENREF_7)^,^ [^9^](#_ENREF_9) Demographic transitions, longevity, and trends of increasing burden in India call for greater attention to rehabilitation services to improve functional outcomes and quality of life among stroke survivors.

Stroke is the most common cause of cognitive and motor impairments. Among the cognitive deficits include a disorder of spatial awareness known as Unilateral Spatial Neglect (USN). USN is a deficit of attention and is described as the inability to report, respond, or orient to sensory or visual stimuli when it appears on the side opposite to the lesion.[^10^](#_ENREF_10) USN is a disorder which can reduce a person’s ability to look, listen or make movements towards one half of their environment. This can also affect their ability to perform many daily functions, such as eating, reading and dressing. Up to 90% of the post-stroke survivors may experience the spatial neglect.[^11^](#_ENREF_11)

USN is a strong predictor of poor motor recovery and functional performances among the stroke subjects.[^12^](#_ENREF_12) The impaired ability has significant physical, social, and emotional implications. Considering the International Classification of Functioning, Disability and Health, USN undoubtedly affects body structure and function that subsequently leads to activity limitation and participation restriction.

Cognitive rehabilitation has been recommended as one of the important areas of stroke research that warrant independent synthesis of evidence generated in LMICs.[^13^](#_ENREF_13) Further, globally, research on cognitive impairment after stroke is at an earlier stage of evolution than research in motor recovery.[^14^](#_ENREF_14) The studies to enhance cognitive deficits among stroke survivor are recommended as a top first question for research.[^15^](#_ENREF_15) The proposed study may be applicable to the *National Stroke Registry Program (NSRP) / National Programme for Prevention and Control of Cancer, Diabetes, Cardiovascular Diseases and Stroke* (NPCDCS) in terms of rehabilitation services to control and prevent disability for comprehensive management of stroke patients.[^16^](#_ENREF_16)^,^ [^17^](#_ENREF_17)

[^15^](#_ENREF_15)

1. **Literature review:**

USN is a frequent and disabling impairment after stroke. The terms unilateral neglect, visuospatial neglect, hemineglect and spatial neglect are used interchangeably. Although the USN usually occurs on the contralesional side in the right brain damage, the deficit may be observed on the ipsilesional side as well as among subjects with the left brain damage.[^11^](#_ENREF_11)^,^ [^18^](#_ENREF_18)^,^ [^19^](#_ENREF_19) The functional performance of an individual with USN gets hampered. Most of the investigations have been focused on the impairment and management of upper limb functions. In addition to this, the USN affects lower limb functions such as locomotion and stair climbing. The post-stroke subjects with USN are at more risk of falling in comparison to the subjects without USN.[^20^](#_ENREF_20)^,^ [^21^](#_ENREF_21) The functional ambulation including stair climbing gets more impaired than the stroke subjects without USN.[^20^](#_ENREF_20)^,^ [^22^](#_ENREF_22) Further, USN has been identified as a key element that may impede upper limb and lower limb recovery.[^12^](#_ENREF_12)

The management of USN poses challenges in clinical practice, affecting the overall recovery. A wide range of therapeutic approaches has been applied in managing USN, including cognitive, behavioural, and pharmacological therapy.[^23^](#_ENREF_23) However, there is no consensus as to which methods are most effective. Cognitive rehabilitation for USN comprises restitutive and compensatory methods. Techniques using the restitutive approach aim to alter the underlying cognitive impairment. Compensatory techniques include teaching strategies to make behavioural adjustments. The emphasis in compensatory strategies is on coping with and finding ways of adapting to existing impairments. In the recent Cochrane review by *Bowen A et al (2013)*[^11^](#_ENREF_11) it has been concluded that the effectiveness of cognitive rehabilitation interventions for reducing the disabling effects of neglect and increasing independence remains unproven. No cognitive rehabilitation approach can be supported or refuted based on the current evidence. Though no specific technique has been found to be strongly evident for reducing USN, the activity based methods has been recommended to enhance functional performances.[^10^](#_ENREF_10)

Mirror therapy is an evident method for enhancing sensori-motor recovery in poststroke hemiparesis.[^24^](#_ENREF_24) The technique utilizes the mirror-illusion created by the movement of sound limb that is perceived as the paretic limb. The technique is a simple and economical method that can stimulate the brain noninvasively. The intervention unquestionably has neural foundation. Multiple brain areas such as occipital lobe dorsal frontal area, and corpus callosum are involved during the mirror therapy. Bilateral premotor, primary motor cortex, primary somatosensory cortex, and cerebellum reorganize enhancing the function of damaged brain. The motor areas of the lesioned-hemisphere receive visuo-motor processing information through parieto-occipital lobe. Mirror neurons (a set of cells in premotor cortex and inferior parietal lobule activates during observation as well as performing a movement) may also play a possible role in the cortico-mechanism out of mirror therapy.[^25^](#_ENREF_25) The therapy may positively affect body representation in post-stroke subjects with motor impairment. In an investigation carried out by *Tosi G et al (2017)*[*^26^*](#_ENREF_26) on a group of 45 post-stroke patients, following the mirror therapy session bisection scores shifted distally, compatibly with a partial correction of the metric representation of that arm. The effects showed some variability with the laterality of the lesion and the duration of the illness.

*Pandian JD et al (2014)*[*^27^*](#_ENREF_27) in their RCT investigated the effect of mirror therapy on USN among 48 acute (48 hours) post-stroke subjects (thalamic and parietal lobe lesions). The study demonstrated favorable results in favor of mirror therapy (12 hours, 5 days/ week for a month) for reducing USN as measured by star cancellation, line bisection, and picture identification task at 6-month. Only acute stroke, specific area of lesions, and lack of USN-specific functional measure are some of the noticeable weakness of the study. Further, the associated motor recovery was also not considered. In experimental protocol, the subjects were provided only movements as mirror therapy rather than using meaningful tasks as media. *Thieme et al (2012)*[*^28^*](#_ENREF_28) in their RCT among acute stroke (within 3 months) using mirror therapy found positive effect for USN; however, on a small subsample of 3 patients. Other than the small sample, the secondary objective and chronicity are also the major weakness of the study. *Dohle C et al (2009)*[*^29^*](#_ENREF_29)also conducted an RCT using mirror therapy (1/2 hour, 5 days/week for 6 weeks) on 36 acute stroke (within 8 weeks) with middle cerebral artery involvement. In the study, a sub-sample of 20 subjects had USN, the 11 experimental subject exhibited positive effect of mirror therapy. However, the investigator considered the improvement of hemineglect as a positive side effect whose independent therapeutic value remains to be proven. Further studies are recommended to explore the interplay between recovery in the USN and sensori-motor domain.

Mirror has also been recommended to be used a feedback tool among USN subjects. *Ramachndran et al (1999)*[*^30^*](#_ENREF_30) has proposed and tested the position of a mirror on the patient’s right side in the parasagittal plane, so that when the patient rotates his head rightward and looks into the mirror, he sees the neglected side of the world reflected in the mirror. They argued that since the sensory information was coming from the left side, this would make him to overcome the neglect. In their pilot experiments, the presence of the mirror enhanced the patients’ awareness of the neglected field, so that they reached correctly for an object that was shown in the neglected field.

The magnification of vision may increases the cortical representation of the body part. It has been proposed that magnification of a body part might unmask pre-existing connections between cortical areas or disinhibition of pre-existing synapses and the activation of previously silent brain networks.[^31^](#_ENREF_31) *Ambron et al (2018)*[*^32^*](#_ENREF_32) investigated the effect of hand-motor tasks under magnified vision condition on 25 chronic stroke subjects (> 6 months). Twenty-eight percent of individuals showed an immediate significant improvement averaged across all tasks with magnification; similar beneficial responses were also observed in 32% of individuals after a short delay. The results suggested that magnification of the image of the hand may be of utility in rehabilitation of individuals with stroke.

***Rationale:*** Cognitive deficits are a major cause of disability in post- stroke subjects. Spatial neglect has visual, perceptual, and motor factors. Post-stroke with USN exhibited poor functional outcome when compared with individuals without USN. The presence of USN is associated with poor upper limb motor control, both at subacute and chronic stages.[^12^](#_ENREF_12) A range of therapeutic approaches comprising cognitive and behavioral approaches has been applied in visuospatial neglect. However, there is no consensus as to which rehabilitation method is most effective.[^23^](#_ENREF_23) There is very limited evidence that cognitive rehabilitation may have an immediate beneficial effect on neglect. Further, there is a lack of investigations and insufficient evidence related to upper limb therapies in post-stroke subject with USN. This justifies the need for further clinical trials of cognitive rehabilitation for neglect.[^11^](#_ENREF_11) The investigations on USN using mirror therapy have either been conducted on acute subjects or underestimating the importance of motor outcome. The common neurobiological mechanism for proportional recovery rule[^33^](#_ENREF_33) both for the upper limb and USN demands the intervention of dual action. In addition to the upper limb, the USN also affects the motor and functional performance of the lower limb. However, no USN rehabilitation techniques have incorporated the lower limb aspect. The other crucial knowledge gaps in this context are: use of magnified vision for USN and use of mirror in the parasagital plane for USN. It is understood that appropriate and comprehensive intervention for USN would not only reduce the cognitive disability but also induce substantial motor and functional recovery.

1. **Novelty:**

The proposed study would be the first investigation to utilize the tasks for providing the mirror therapy among stroke subjects with USN. The meaningful tasks induce movements that appear out of an interaction between multiple brain areas to manipulate a real-world object. Such movements increase the cortical activity and may reduce greater the deficit than the movements without task.[^34^](#_ENREF_34)

Apart from the upper limb, the activity-based mirror therapy protocol[^35^](#_ENREF_35) will also be provided for the lower limb to comprehensively reduce the impact of USN. This would be the first study to incorporate the lower limbs in any USN rehabilitation study.

Magnifying the image of a body part (hand or arm), has a positive and beneficial effect on activity performance and thus motor recovery among stroke subjects.[^32^](#_ENREF_32)^,^ [^36^](#_ENREF_36) Mirror therapy providing image without magnification has been utilized in management of USN. No cognitive rehabilitation protocol utilizing magnified image of the neglected body part has been investigated yet. The proposed study would be the first investigation of magnified mirror for creating illusion for the affected upper limb by the less-affected side. When the vision of the limb will be magnified, the illusion may induce enlargement of the cortical representation of the body part. Thus, enhancing the underlying neuromechanism[^25^](#_ENREF_25) of mirror therapy.

The fourth novelty of this proposed investigation is the use of mirror therapy to provide visual feedback for the affected limb (upper or lower limb) in the parasagital plane. Although it has been suggested by the developers of the mirror therapy[^30^](#_ENREF_30), no mirror-based rehabilitation trials has applied the concept of visual feedback for USN.

Further, the present study will also be focused on management of USN among chronic stroke[^37^](#_ENREF_37), unlike the previous investigations.

1. **Study objectives**:

**Primary objectives**

- 1. To determine the effectiveness of Task-based and Magnified mirror therapy on Unilateral Spatial Neglect (USN) among post-stroke hemiparetic subjects
  2. To determine the effectiveness of the protocol on motor recovery of the subjects

**Secondary objectives**

- 1. To determine the effectiveness of the Task-based and Magnified mirror therapy on functional performance of the subjects
  2. To determine the effectiveness of the protocol on disability status among the subjects.

1. **Methodology:**

**Hypothesis:**

1. Task-based and Magnified Mirror therapy protocol for the paretic upper limb provided for 2 months will decrease the Unilateral spatial neglect by 50% in 1 to 36 months post stroke subjects with hemiparesis when compared with the conventional management.
2. Task-based and Magnified Mirror therapy protocol for the paretic upper limb provided for 2 months will enhance the motor recovery by 30% in 1 to 36 months post stroke subjects with hemiparesis when compared with the conventional management.

**Design***:* Randomized controlled, single blinded trial

**Setting:** Neuro-Rehab Laboratory*,* Department of Occupational Therapy, Pt. Deendayal Upadhyaya National Institute for Persons with Physical Disabilities, New Delhi

**Inclusion Criteria:**

- Age: 20 to 80 years
- Both male & female
- First episode of unilateral stroke (as defined by WHO)
- Ischemic or hemorrhagic stroke
- 1 to 36 months after the stroke onset
- Hemiparesis (right or left) (as assessed by Fugl-Meyer assessment[^38^](#_ENREF_38) upper extremity subsection: 0 to 66)
- USN (defined as an asymmetry between the Letter cancellation task omissions in the contralesional (left) and ipsilesional (right) visual field of at least 2 letters)[^33^](#_ENREF_33)
- Normal visual abilities (with or without glasses)
- Muscle tone: Modified Ashworth scale 1 to 2

**Exclusion criteria**:

- Receptive communication or other language disorder (which could interfere with the assessment and treatment process)
- Severe other cognitive impairment (MMSE ≤ 17)[^39^](#_ENREF_39)
- Homonymous Hemianopia.
- Contractures and deformities of hand / finger
- Use of hand splinting or orthosis
- Complex regional pain syndrome
- Concomitant medical illness
- Cardiovascular instability: resting systolic blood pressure >200mmHg and resting diastolic blood pressure >100 mmHg
- Pregnancy
- Renal infection or failure
- Severe depression (Beck depression inventory[^40^](#_ENREF_40)^,^ [^41^](#_ENREF_41) > 30)

**Sample size:**

The power calculation was done using the values *(Box 1)* for USN assessment from a study on mirror therapy for USN.[^27^](#_ENREF_27) Considering the beta = 0.1 and α = 0.05, the calculation inferred that 37 subjects in each group would be sufficient to detect the desired change. However, to compensate possible drop outs (15%), 43 subjects in each group will be enrolled. Thus, the total sample size will be **86**.

*Box 1*

| Study | Primary measure | Values used | Alpha | Beta | Estimated sample size each group | Total sample size |
| --- | --- | --- | --- | --- | --- | --- |
| *Pandian JD et al*[*^27^*](#_ENREF_27) | Line Bisection Test | Mean difference = 8.6  SD = 10.6 | 0.05 | 0.1 | 37 | 74 |

**Group Allocation and Concealment:** The subjects will be randomly allocated between the experimental and control groups in block of 10. The assessor will be unaware about the group allocation of the subjects and will apply the outcome measures both pre- and post intervention. The randomization process will be conducted by an office staff, not associated with the study, using the SPSS Version 23 software. The intervention will be allocated in the ratio of 1:1. The assignment will be serially arranged in sealed opaque envelopes. The subjects will also be blinded to the purpose of the experimental intervention.

**Intervention:**

*Experimental group:* 40 sessions, 5/week, during 2 months, 1 hour experimental intervention in addition to 1 hour control motor rehabilitation.[^42^](#_ENREF_42)

*Control group Intervention:* Conventional motor rehabilitation (2-hour, 40 sessions).

*Rest Period:* A 10-minute rest period will be provided after 1 hour session to all the study participants. The subjects will be allowed to have water or light snacks during the break.

*Assessment Sessions: 02 pre-, 01 post- intervention and 01 follow-up (1-month)*

**Experimental Intervention:**

**T**ask-based and **MAG**nified **M**irror Therapy for **U**nilateral **S**patial **N**eglect (T-MAGUSN)

The experimental intervention will comprise of the following components -

- 1. Task-based upper limb mirror therapy (20 minutes x 40 sessions)
  2. Task-based lower limb mirror therapy (20 minutes x 40 sessions)
  3. Magnified mirror therapy (10 minutes x 40 sessions)
  4. Task-based parasagital plane visual feedback mirror therapy (10 minutes x 40 sessions)
     1. Task-based upper limb mirror therapy

The protocol guidelines will be followed as per our previous work[^34^](#_ENREF_34) on upper limb motor recovery using task-based mirror therapy. However, necessary modification in terms of USN as an objective of the intervention will be carried out. A wooden mirror box / frame of size (LxBxH = 30x18x40 inches; reaching height and breadth) will be used. Various objects such as pegs, pyramids, blocks, clay, ball, U-clips, balls and coins will be utilized to provide the mirror therapy. The textured objects[^24^](#_ENREF_24) will also be provided to provide the multi-sensory awareness. The subject will be made to sit close to a table on which the mirror box / frame will be placed vertically at the mid sternum level. The less-affected upper limb will be placed in front of the mirror to visualize the image while the affected limb will be placed inside the mirror box. Subject could perceive the image of the less-affected limb as the affected limb. By observing the image of the less-affected upper extremity as a projection over the affected limb, an illusion will be created *(figure 1).* The tasks will be provided with an objective to perceive the body part, movement and object in the ipsilesional space as the illusion of the contralesional space. The objects will be reached for, grasped, lifted, placed and manipulated in different spatial planes (Forward/backward, superior/inferior, medially/laterally, diagonally). The therapist will instruct for the direction and may locate the object in the desired plane by standing in front of the patient. The movements of all the joints (shoulder, elbow, forearm, wrist, hand and fingers) will be utilized in view of the associated motor impairment. The number of tasks, joints, repetition, speed, time and resistance will be considered as parameters for grading the intervention.

*
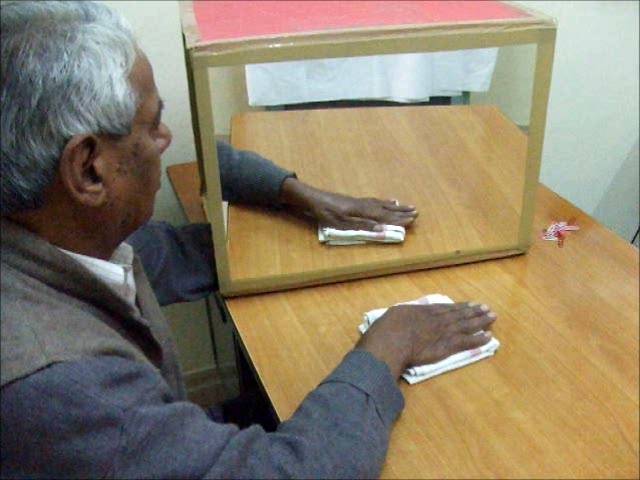
*

*Figure 1: A left hemiparetic subject with USN performing wiping task by the right upper limb; experiencing the illusion for the left upper limb (in the contralesional space) created by the functional use of the right side.*

- - 1. *Task-based lower limb mirror therapy*

For the lower limb mirror therapy, the protocol will be followed as per our previous study.[^35^](#_ENREF_35) A short-sitting mirror frame measuring 24 x 72 inches will be used with a provision of the mirror to be tilted in the sagittal plane to view the mirror at an acute angle, between 75 and 85 degrees. The arrangement enabled maximum mirror perception of the moving limb without having any possible sight of the body part. Another frame measuring 36 x 48 inches will be used the long-sitting MT. The subject will receive task-based movements such as pedaling, wiping, and shifting pillow were provided on the less-affected side in front of the mirror while hiding the affected limb *(figure 2).* The activities will be graded as described for the upper limb program.

**
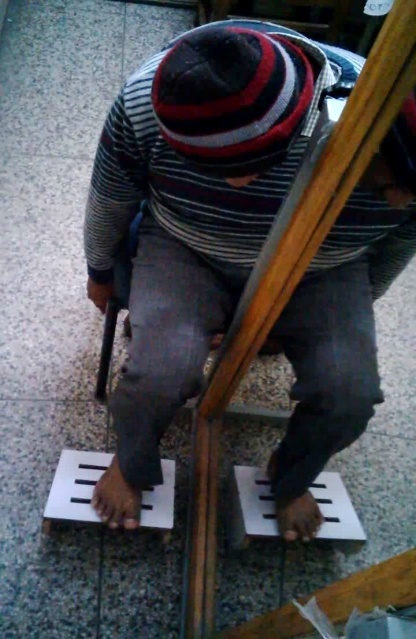
**

*Figure 2: A left hemiparetic subject with USN performing Rocking-board activity by the right upper limb; experiencing the illusion for the left lower limb (in the contralesional space) created by movement of the right side.*

- - 1. *Magnified mirror therapy*

To provide the intervention a magnified (2-3X) mirror *(figure 3)* will be utilized. The task-based therapy, guidelines as described at *above 1*, will be followed. The hand and finger manipulation using various meaningful objects will be used by the less-paretic hand to provide the illusion for the paretic hand. The subject will be asked to visualize the magnified body part and objects minutely and then manipulate the objects. The movements will be performed indifferent spatial planes. The number of tasks, repetition, speed, time and resistance will be considered as parameters for grading the intervention.


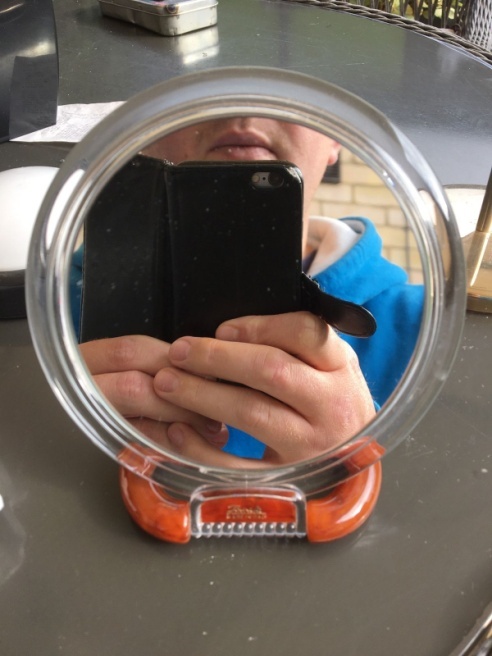


*Figure 3: A magnified mirror*

- - 1. *Task-based parasagital plane visual feedback mirror therapy*

A mirror frame will be positioned vertically on the less-affected side with a therapist standing behind it. The mirror frame will be held parallel to the sagittal plane so that its right edge will be close to the patient’s paretic shoulder. The subject will be asked to turn his head and eyes to look into the center of the mirror so he could clearly see the mirror reflection of the people/ objects that are on the left side. To ensure that the patient is looking in the mirror, the therapist (standing behind the mirror) will enquire about holding of the mirror. A second therapist standing on the patient’s paretic side will hold a block or cone and move towards the subject so that it is well within the reach of the subject’s non-paretic hand but entirely within the neglected (paretic) visual field – about 8 inches below and to the paretic side nose. The therapist will then enquire about the object and ask the patient to reach for the item. Similarly, the objects will be positioned and enquired and asked for the reach and manipulation in different locations. The number of objects, repetition, speed, time and resistance will be considered as parameters for grading the intervention.

**Control intervention**

In addition to the conventional motor rehabilitation,[^42^](#_ENREF_42) the control group will be provided following intervention[^43^](#_ENREF_43) for the USN rehabilitation for a dose match (2- hour) program –

- Mirror frame will be placed in front of the patient while he will be performing activities of daily living, ambulation and motor activities (unilateral and bilateral) in order to draw attention to the neglected side.
- Mid-line crossing activities – unilateral and bilateral
- Scanning, puzzle, trail making, drawing activities

**Outcome measures:**

**Primary measures**

**Unilateral Spatial Neglect**

1. ***Line Bisection Test (LBT)***

There are three 20-cm horizontal black lines in the LBT. One line is on the right of the page, one line is in the center, and one is on the left. The patients will be instructed to mark the center of each line. Errors will be measured in centimeters from the true midline. Leftward errors will be indicated with negative numbers and rightward errors will be indicated with positive numbers. The cutoff score for unilateral visual neglect will be considered as an error of more than 1.4 cm left or right. It is a reliable and valid measure of USN and commonly used measure in stroke studies.[^27^](#_ENREF_27)

1. ***Letter Cancellation Test (LCT)***

A single-target Letter Cancellation Test (LCT) will be the main outcome measure to assess USN requested to cross all O’s on an A4 paper taped on the table and aligned to their sagittal midline. Each paper contained 40 O’s (20 in the contralesional and 20 in the ipsilesional visual field, respectively left and right) and 425 distractor letters. USN has been defined as an asymmetry between the LCT omissions in the contralesional (left) and ipsilesional (right) visual field of at least 2. It also possesses adequate psychometric properties and usually applied in stroke investigations.[^33^](#_ENREF_33)

**Motor Recovery**

***Fugl-Meyer Assessment***

The Fugl-Meyer assessment[^38^](#_ENREF_38) (FMA) is a performance-based measure. It has five sections to test a specific construct (motor, balance, sensation, range of motion and pain). The motor section of FMA is arranged hierarchically and evaluates aspects of movement, reflexes, coordination, and speed. The motor section is further divided into two subsections; the upper extremities (upper arm and wrist and hand) and lower extremities. The upper extremity (FMA-UE) subsection will be used to assess upper extremity recovery in the present study. It is scored out of 66, with sub-score of 36 for the upper arm (FMA-UA) and 30 for the wrist and hand (FMA-WH). Most of the items are scored on a 3-point ordinal scale; from 0 – no function to 2 – full function. The lower-extremity subsection (FMA-LE) comprised items ranging from reflexes, synergy, combining synergies, out of synergy, and coordination. FMA-LE has a total of 17 items, which are scored on a 3-point ordinal scale (0 [no performance] to 2 [full performance]). The FMA has been found to be a highly reliable (r = 0.98–0.99) and valid (r = 0.61–0.94) tool to assess the motor recovery of post stroke patients.[^44-46^](#_ENREF_44)

**Secondary measures**

- - 1. ***Catherine Bergego Scale***

The Catherine Bergego Scale (CBS) is used to measure neglect. It is a valid, reliable, and sensitive measure of poststroke neglect. Specifically, it has good internal consistency, inter-rater reliability, concurrent validity and convergent validity. The CBS is a functional neglect assessment because it assesses neglect through observation of activities such as eating and locomotion (i.e. collisions, navigation items). The CBS has items that assess for personal, peripersonal, and extrapersonal neglect and the type of neglect each item assesses has been delineated. Assessors score 10 items using a 4-point scale. Higher CBS scores (range: 0-30) indicate more severe neglect. Kessler Foundation Neglect assessment Process guidelines will be used to apply the CBS.[^47^](#_ENREF_47)^,^ [^48^](#_ENREF_48)

- - 1. ***Berg Balance Scale***

The Berg Balance Scale (BBS) has been developed to measure balance among older people with impairment in balance function by assessing the performance of functional tasks. It is a valid instrument used for evaluation of the effectiveness of interventions and for quantitative descriptions of function in clinical practice and research.[^49^](#_ENREF_49) It is a commonly used measure to assess balance ability among post-stroke subjects.[^50^](#_ENREF_50) It is a 14-item measure, items ranging from sit-to-stand to standing on one foot. All the items are score on 0 to 4, 4 being the positive performance. The total score is 56, with < 40 and < 20 being considered as medium and high fall risk, respectively for the subject.

- - 1. ***Functional Ambulation Category (FAC)***

Functional Ambulation Category (FAC) measures the walking ability of the poststroke subjects. It is an economical and simple method to quantify independency in ambulation. The scale defines 6 categories ranging from 0 (unable to walk) to 5 (independent walking). FAC has exhibited acceptable reliability and validity.[^51^](#_ENREF_51)^,^ [^52^](#_ENREF_52)

- - 1. ***Modified Rankin scale***

The modified Rankin scale (mRS) is a clinician-reported and recommended measure of global disability.[^53^](#_ENREF_53) It is widely used for evaluating trial outcomes of stroke patients and is an end point in randomized clinical trials.[^54^](#_ENREF_54) mRS scores range from 0 (no symptoms at all) to 5 (severe disability)

*Distribution of Outcome measure as per International classification of Functioning (ICF)*

| **Outcome Measure** | **Level of Measure**  (International Classification of Functioning) |
| --- | --- |
| Line Bisection Test (LBT)  Letter Cancellation Test (LCT) | Spatial ability **(Body function)** |
| Fugl-Meyer assessment (FMA) | Motor recovery **(Body function)** |
| Catherine Bergego Scale | Spatial related functions **(Activity)** |
| Berg Balance Scale | Balance **(Activity)** |
| Functional Ambulation Classification (FAC) | Ambulation **(Activity)** |
| Modified Rankin Scale (mRS) | Disability **(Participation)** |

**Data Collection:**

The data will be collected using the assessment proforma provided as ***Annexure I.*** The proforma comprises items/questions related to demographic aspects of the subjects such as age, gender, type of stroke, duration of stroke, area of involvement, functional performance. Additionally, the clinical evaluation of a standard neuro-rehabilitation condition will also be covered. All the standardized outcome measures in form of data collecting tool have been provided in the ***Annexure II to VII.***

The flow of study process and schedule of enrolment, intervention, and assessment (SPIRIT guidelines[^55^](#_ENREF_55)) are provided below –

Post- stroke patients (1 to 36 months) – Screened for the eligibility (n=….)

Block Randomization (n=86)

Conventional Rehabilitation –

4 weeks

Experimental intervention (n=43)

(Task-based & Magnified Mirror Therapy) + Conventional Rehabilitation

40 sessions, 2 hours each during 8 weeks

Control Intervention (n=43)

(Conventional Rehabilitation)

Dose matched for duration

40 sessions, 2 hours each during 8 weeks

Enrolment

Allocation

Follow-up

Post intervention Assessment (T2)

Baseline Assessment (T1)

Follow up Assessment (T3)

**Study Flow Diagram**

Schedule of enrolment, interventions, and assessment –

**SPIRIT Guidelines**

|  | **STUDY PERIOD** | | | | |
| --- | --- | --- | --- | --- | --- |
|  | **Enrolment** | **Allocation** | **Intervention** | | **Follow-up** |
| **TIMEPOINT** | ***-t_1_*** | **0** | ***t_1_*** | ***t_2_*** | ***t_3_*** |
| **ENROLMENT:** |  |  |  |  |  |
| **Eligibility screen** | X |  |  |  |  |
| **Informed consent** | X |  |  |  |  |
| ***Randomization*** |  | X |  |  |  |
| **Allocation** |  | X |  |  |  |
| **INTERVENTIONS:** |  |  |  |  |  |
| ***[Task-based & Magnified Mirror Therapy]*** |  |  |  |  |  |
| ***[Control]*** |  |  |  |  |  |
| **ASSESSMENTS:** |  |  |  |  |  |
| ***[Demographic Information]*** |  | X |  |  |  |
| ***[Patient Characteristic]*** |  | X |  |  |  |
| ***[Standard Neuro-Rehab. Clinical Evaluation]*** |  | X |  |  |  |
| ***LCT*** | X |  |  | X | X |
| ***LBT*** |  |  | X | X | X |
| ***FMA*** |  |  | X | X | X |
| ***CBS*** |  |  | X | X | X |
| ***BBS*** |  |  | X | X | X |
| ***FAC*** |  |  | X | X | X |
| ***mRS*** |  |  | X | X | X |

T1 baseline assessment, T2 assessment after 8 weeks of intervention, T3 assessment after four-week follow-up (12 weeks after start of intervention). LCT = Letter Cancellation Task, LBT = Line Bisection Test, FMA = Fugl-Meyer assessment, CBS = Catherine Bergego Scale, BBS = Berg Balance Scale, FAC = Functional Ambulation Category, mRS= modified Rankin scale

**Data Analysis:**

The data will be analyzed by using IBM SPSS version 23.0. The demographic and baseline features of the study participants will be analyzed in form of mean (SD) / median (IQR) / n (%) and appropriate test [(Mann-Whitney U (U) / independent t (t) / chi-square (χ^2^)] tests will be used to analyze the difference for the characteristics between the groups. For inferential statistics, an intention-to-treat analysis method will be used by carrying forward the last observation. A repeated-measures 2-way ANOVA (continuous data; within factor, time; between factor, group) will be used to study difference for the post intervention and follow-up scores between the groups. The pre-intervention score will be considered as the covariate with group as the independent variable and posttest score as the dependent variable. The significance level will be set at *P*<.05. The dummy tables to represent the findings are provided below -

**Dummy Tables**

**Table 1: Demographic and Clinical characteristics of the participants**

| **S. No.** | **Characteristic** | **Experimental group**  **(n=43)** | **Control group**  **(n=43)** | **Test Statistics** |
| --- | --- | --- | --- | --- |
|  | Age (years) *Mean+SD* |  |  | t:p = |
|  | Time since stroke (months) *Mean+SD* |  |  | t:p = |
|  | Male/female - *n (%)* |  |  | χ^2^:p = |
|  | Socio-economic status (BPL/LIG/MIG/HIG) - *n (%)* |  |  | χ^2^:p = |
|  | Educational qualification (years)  *Mean+SD* |  |  | t:p = |
|  | Marital status (married/unmarried/widowed) - *n (%)* |  |  | χ^2^:p = |
|  | Ischemic/Hemorrhagic - *n (%)* |  |  | χ^2^:p = |
|  | Area of involvement (Frontal/parietal/frontoparietal/internal capsule/ basal ganglia/thalamus/ multiple/others) - *n (%)* |  |  | χ^2^:p = |
|  | Risk factors – *n (%)* |  |  | χ^2^:p = |
|  | 1. Hypertension - *n (%)* 2. Smoking - *n (%)* 3. Alcoholic - *n (%)* 4. Tobacco Use - *n (%)* 5. Hereditary - *n (%)* 6. Diabetes mellitus - *n (%)* 7. Obesity- *n (%)* 8. Pregnancy - *n (%)* 9. Coronary artery disease - *n (%)* 10. Peripheral vascular disease - *n (%)* 11. Dyslipidemia- *n (%)* 12. Previous Stroke - *n (%)* |  |  | χ^2^:p =  χ^2^:p =  χ^2^:p =  χ^2^:p =  χ^2^:p =  χ^2^:p =  χ^2^:p =  χ^2^:p =  χ^2^:p =  χ^2^:p =  χ^2^:p =  χ^2^:p = |
|  | Side of involvement (Right/left) – *n (%)* |  |  | χ^2^:p = |
|  | Dominant side (Right/left) – *n (%)* |  |  | χ^2^:p = |
|  | BRS-U (Stage1/2/3/4/5/6) – *n (%)* |  |  | χ^2^:p = |
|  | BRS-H (Stage 1/2/3/4/5/6) – *n (%)* |  |  | χ^2^:p = |
|  | BRS-L (Stage 1/2/3/4/5/6) – *n (%)* |  |  | χ^2^:p = |
|  | Shoulder subluxation (Grade 1/2/3) – *n (%)* |  |  | χ^2^:p = |
|  | Shoulder support– *n (%)* |  |  | χ^2^:p = |
|  | Hand Splint– *n (%)* |  |  | χ^2^:p = |
|  | MAS (0/1/1+/2) – *n (%)*   1. Shoulder adductors 2. Elbow flexors 3. Forearm pronators 4. Wrist flexors |  |  | χ^2^:p =  χ^2^:p =  χ^2^:p =  χ^2^:p = |
|  | Walking devices (walker/quadripod/stick) – *n (%)* |  |  | χ^2^:p = |
|  | AFO (hinged/non-hinged) – *n (%)* |  |  | χ^2^:p = |
|  | Pain (VAS)   1. Shoulder *(Median; IQR)* 2. Wrist *(Median; IQR)* 3. Hand *(Median; IQR)* |  |  | U:p=  U:p=  U:p= |
|  | Cognitive impairment (mild/moderate) *- n (%)* |  |  | χ^2^:p = |
|  | Psychosocial behavior (depression/aggression) - *n (%)* |  |  | χ^2^:p = |
|  | Speech (Expressive aphasia/dysarthria) - *n (%)* |  |  | χ^2^:p = |
|  | Language impairment (writing/reading) - *n (%)* |  |  | χ^2^:p = |

BPL: Below poverty line, LIG: Lower income group, MIG: Middle income group, HIG: Higher income group, UG: Under graduate, PG: Post graduate, BRS-U: Brunnstrom recovery stage of arm, BRS- H: Brunnstrom recovery stage of hand, BRS- L: Brunnstrom recovery stage of lower limb SD: Standard deviation , MAS- Modified ashworth scale, IQR – Inter quartile range, AFO-Ankle foot orthosis, VAS – Visual analog Scale

**Table 2: Changes in Pre- and Post-intervention, and Follow-up scores of the Letter Cancellation Task (LCT) between the experimental and control groups**

| **Outcome measure** | **Pre-intervention** | | **Post-intervention (8-week)** | | **Follow-up (12-week)** | |  | | |
| --- | --- | --- | --- | --- | --- | --- | --- | --- | --- |
|  | **Experimental Group (n=43)** | **Control Group (n=43)** | **Experimental Group (n=43)** | **Control**  **Group (n=43)** | **Experimental Group (n=43)** | **Control**  **Group (n=43)** | **Difference between the means**  **(95% CI)** | **F** | **P value** |
| **LCT (Right)**  *Mean*±*SD*  *Maximum score = 20* |  |  |  |  |  |  |  |  |  |
| **LCT (Left)**  *Mean*±*SD*  *Maximum score = 20* |  |  |  |  |  |  |  |  |  |

n – Number, SD – standard deviation, CI - confidence interval, F – test value ANOVA

**Table 3: Changes in Pre- and Post-intervention, and Follow-up scores of the Line Bisection Test (LBT) between the experimental and control groups**

| **Outcome measure** | **Pre-intervention** | | **Post-intervention (8-week)** | | **Follow-up (12-week)** | |  | | |
| --- | --- | --- | --- | --- | --- | --- | --- | --- | --- |
|  | **Experimental Group (n=43)** | **Control Group (n=43)** | **Experimental Group (n=43)** | **Control**  **Group (n=43)** | **Experimental Group (n=43)** | **Control**  **Group (n=43)** | **Difference between the means**  **(95% CI)** | **F** | **P value** |
| **LBT (Right)**  *Mean*±*SD*  *Maximum score = 20* |  |  |  |  |  |  |  |  |  |

n – Number, SD – standard deviation, CI - confidence interval, F – test value ANOVA

**Table 4: Changes in Pre- and Post-intervention, and Follow-up scores of the Fugl-Meyer Assessment (FMA) between the experimental and control groups**

| **Outcome measure** | **Pre-intervention** | | **Post-intervention (8-week)** | | **Follow-up (12-week)** | |  | | |
| --- | --- | --- | --- | --- | --- | --- | --- | --- | --- |
|  | **Experimental Group (n=43)** | **Control Group (n=43)** | **Experimental Group (n=43)** | **Control**  **Group (n=43)** | **Experimental Group (n=43)** | **Control**  **Group (n=43)** | **Difference between the means**  **(95% CI)** | **F** | **P value** |
| **FMA**  *Mean*±*SD*  *Maximum score = 100* |  |  |  |  |  |  |  |  |  |
| **FMA-UE**  *Mean*±*SD*  *Maximum score = 66* |  |  |  |  |  |  |  |  |  |
| **FMA-UA**  *Mean*±*SD*  *Maximum score =36* |  |  |  |  |  |  |  |  |  |
| **FMA-WH**  *Mean*±*SD*  *Maximum score =30* |  |  |  |  |  |  |  |  |  |
| **FMA-LE**  *Mean*±*SD*  *Maximum score = 34* |  |  |  |  |  |  |  |  |  |

n – Number, SD – standard deviation, CI - confidence interval, F – test value ANOVA, UE-upper extremity, UA-upper arm, WH-wrist-hand, LE – Lower extremity

**Table 5: Changes in Pre- and Post-intervention, and Follow-up scores of the Catherine Bergego Scale (CBS) between the experimental and control groups**

| **Outcome measure** | **Pre-intervention** | **Post-intervention (8-week)** | **Follow-up (12-week)** | | | |  | | |
| --- | --- | --- | --- | --- | --- | --- | --- | --- | --- |
|  | **Experimental Group (n=43)** | **Control Group (n=43)** | **Experimental Group (n=43)** | **Control**  **Group (n=43)** | **Experimental Group (n=43)** | **Control**  **Group (n=43)** | **Difference between the means**  **(95% CI)** | **F** | **P value** |
| **CBS**  *Mean*±*SD*  *Maximum score =30* |  |  |  |  |  |  |  |  |  |

n – Number, SD – standard deviation, CI - confidence interval, F – test value ANOVA

**Table 6: Changes in Pre- and Post-intervention, and Follow-up scores of the Berg Balance Scale (BBS) between the experimental and control groups**

| **Outcome measure** | **Pre-intervention** | | **Post-intervention (8-week)** | | **Follow-up (12-week)** | |  | | |
| --- | --- | --- | --- | --- | --- | --- | --- | --- | --- |
|  | **Experimental Group (n=43)** | **Control Group (n=43)** | **Experimental Group (n=43)** | **Control**  **Group (n=43)** | **Experimental Group (n=43)** | **Control**  **Group (n=43)** | **Difference between the means**  **(95% CI)** | **F** | **P value** |
| **BBS** *Mean*±*SD*  *Maximum score =56* |  |  |  |  |  |  |  |  |  |

n – Number, SD – standard deviation, CI - confidence interval, F – test value ANOVA

**Table 7: Changes in Pre- and Post-intervention, and Follow up scores of the Functional Ambulation Category (FAC) between the experimental and control groups**

| **Outcome measure** | **Pre-intervention** | |  | **Post-intervention** | |  | **Follow-up** | | |
| --- | --- | --- | --- | --- | --- | --- | --- | --- | --- |
|  | **Experimental Group (n=43)** | **Control Group (n=43)** | **Test**  **Statistics** | **Experimental Group (n=43)** | **Control**  **Group (n=43)** | **Test**  **Statistics** | **Experimental Group (n=43)** | **Control**  **Group (n=43)** | **Test**  **Statistics** |
| FAC 0 *– n (%)* |  |  | χ^2^:p = |  |  | χ^2^:p = |  |  | χ^2^:p = |
| FAC 1*– n (%)* |  |  |  |  |  |  |  |  |  |
| FAC 2*– n (%)* |  |  |  |  |  |  |  |  |  |
| FAC 3*– n (%)* |  |  |  |  |  |  |  |  |  |
| FAC 4*– n (%)* |  |  |  |  |  |  |  |  |  |
| FAC 5*– n (%)* |  |  |  |  |  |  |  |  |  |

n – Number

**Table 8: Changes in Pre- and Post-intervention, and Follow up scores of Modified Rankin Scale (mRS) between the experimental and control groups**

| **Outcome measure** | **Pre-intervention** | |  | **Post-intervention** | |  | **Follow-up** | | |
| --- | --- | --- | --- | --- | --- | --- | --- | --- | --- |
|  | **Experimental Group (n=43)** | **Control Group (n=43)** | **Test**  **Statistics** | **Experimental Group (n=43)** | **Control**  **Group (n=43)** | **Test**  **Statistics** | **Experimental Group (n=43)** | **Control**  **Group (n=43)** | **Test**  **Statistics** |
| mRS 0 *– n (%)* |  |  | χ^2^:p = |  |  | χ^2^:p = |  |  | χ^2^:p = |
| mRS 1*– n (%)* |  |  |  |  |  |  |  |  |  |
| mRS 2*– n (%)* |  |  |  |  |  |  |  |  |  |
| mRS 3*– n (%)* |  |  |  |  |  |  |  |  |  |
| mRS 4*– n (%)* |  |  |  |  |  |  |  |  |  |
| mRS 5*– n (%)* |  |  |  |  |  |  |  |  |  |

n – Number

**Data management:**

Data from clinical assessments will be recorded in paper booklets for each patient and converted with double data entry into a PC data file stored on a secured server of the site of study. A copy of data will also be stored in a secured pen drive on weekly basis. A data code sheet will be developed, giving a unique code to each information and numerical scoring system for each of them. The data will be entered into Excel spread sheet before the analysis by the SPSS software. The entry will be done on completion of each subject’s pre or post assessment. Random check of the entered data will be conducted by the principal investigator. Participants’ paper files will be kept for storage for a period of 5 years and electronic data will be stored on a secured server. Confidential information will be converted into unique codes and the key to these codes will be kept in a separate file on a password protected electronic location.

**Ethical Consideration:**

For this study, the approval will be sought from the Institutional ethics committee (IEC) of *Pt. Deendayal Upadhya National Institute for Persons with Physical Disabilities, New Delhi, India.* The trial will comply with the principles of the “declaration of Helsinki.” Before the start of participation in this study, written informed consent will be obtained. All data collected during this study will be confidential but with open access for investigators / research staff at the study site. To protect the privacy of the participants, they will be given unique numerical codes. The key to this code will be maintained securely and confidentially by the principal Investigator. No information with which the participant can be identified will be available in any other study-related (electronic) document. All assessment forms, reports, and other (electronic) records will be coded and handled to maintain strict participant confidentiality.

The participants may withdraw from the study at any time without giving an explanation and without any negative consequences for their care or rehabilitation in the future. Based on our previous studies,[^24^](#_ENREF_24)^,^ [^34^](#_ENREF_34)^,^ [^35^](#_ENREF_35) the judgment is that there is no or very low risk of adverse events in this proposed investigation. If any adverse event is observed during the protocol, the same will be reported to the IEC and described in the dissemination of the work.

**Registration:**

The study will be registered at Clinical Trial Registry of India (CTRI).

**Dissemination:**

The findings of the study will be disseminated as-

- - 1. Oral / poster presentation: at National / International conference related to Rehabilitation/Neurology/Occupational therapy.
    2. Publication: in peer reviewed indexed journal of repute (Impact factor >1), related to Stroke Rehabilitation.

The CONSORT (Consolidated Standards of Reporting Trials) guidelines for the Non-pharmacologic treatments will be followed for the reporting of the study findings.[^56^](#_ENREF_56)

1. **Expected outcomes**

This proposed study will lead to development of a novel rehabilitation protocol for the management of USN in stroke. The protocol will reduce the impact of cognitive disability among post-stroke survivors by reducing USN and enhancing the motor and functional recovery. This will reduce the impact of overall stroke disability and enhance the quality of life of the post-stroke survivors. The study, once successful, can be applied for stroke rehabilitation in various institutions, centers, and hospitals across the country improving the health care for the post-stroke survivors.

1. **Limitations of this study**

In view of various factors associated with the stroke, some of possible limitations of this study could be heterogeneity of the potential study subjects in terms of area of brain involvement, type of USN deficit, and motor recovery stages. The results from this study will be unable to determine the level of illusion out of mirror therapy that would reduce the USN.

1. **Future plans based on expected outcomes**

The findings of the present study, if favorable, may be investigated for other neurocognitive deficits among post-stroke patients. The protocol may be refined considering the various types of USN. Further, the USN protocol of mirror therapy may be integrated with already established mirror-based motor regime.

1. **Timelines:**

| **TASK** | Preliminary work done | |  | **Months** | | | | | | | | | | | | | | | | | |
| --- | --- | --- | --- | --- | --- | --- | --- | --- | --- | --- | --- | --- | --- | --- | --- | --- | --- | --- | --- | --- | --- |
|  |  |  | 00 | 02 | 04 | 06 | 08 | 10 | 12 | 14 | 16 | 18 | 20 | 22 | 24 | 26 | 28 | 30 | 32 | 34 | 36 |
| Development and refinement of the protocol |  |  |  |  |  |  |  |  |  |  |  |  |  |  |  |  |  |  |  |  |  |
| Enrolment & Pre-assessment |  |  |  |  |  |  |  |  |  |  |  |  |  |  |  |  |  |  |  |  |  |
| Providing experimental + conventional m/m |  |  |  |  |  |  |  |  |  |  |  |  |  |  |  |  |  |  |  |  |  |
| Post- & follow up assessments |  |  |  |  |  |  |  |  |  |  |  |  |  |  |  |  |  |  |  |  |  |
| Analysis Pre-Post-follow up intervention |  |  |  |  |  |  |  |  |  |  |  |  |  |  |  |  |  |  |  |  |  |
| Reporting & Dissemination |  |  |  |  |  |  |  |  |  |  |  |  |  |  |  |  |  |  |  |  |  |

**References**

1. World health report – 2002, from the world health organization.

2. <http://ncdirindia.org/stroke/BS_FactSheet_Stroke.aspx>.

3. Banerjee TK, Das SK. Fifty years of stroke researches in india. *Ann Indian Acad Neurol*. 2016;19:1-8

4. Thrift AG, Thayabaranathan T, Howard G, Howard VJ, Rothwell PM, Feigin VL, Norrving B, Donnan GA, Cadilhac DA. Global stroke statistics. *Int J Stroke*. 2017;12:13-32

5. Dalal PM, Malik S, Bhattacharjee M, Trivedi ND, Vairale J, Bhat P, Deshmukh S, Khandelwal K, Mathur VD. Population-based stroke survey in mumbai, india: Incidence and 28-day case fatality. *Neuroepidemiology*. 2008;31:254-261

6. Mishra NK, Khadilkar SV. Stroke program for india. *Ann Indian Acad Neurol*. 2010;13:28-32

7. Pandian JD, Sudhan P. Stroke epidemiology and stroke care services in india. *J Stroke*. 2013;15:128-134

8. Kamalakannan S, Gudlavalleti ASV, Gudlavalleti VSM, Goenka S, Kuper H. Incidence & prevalence of stroke in india: A systematic review. *Indian J Med Res*. 2017;146:175-185

9. Sureshkumar K, Murthy GV, Kinra S, Goenka S, Kuper H. Development and evaluation of a smartphone-enabled, caregiver-supported educational intervention for management of physical disabilities following stroke in india: Protocol for a formative research study. *BMJ Innov*. 2015;1:117-126

10. Liu KPY, Hanly J, Fahey P, Fong SSM, Bye R. A systematic review and meta-analysis of rehabilitative interventions for unilateral spatial neglect and hemianopia poststroke from 2006 through 2016. *Arch Phys Med Rehabil*. 2019;100:956-979

11. Bowen A, Hazelton C, Pollock A, Lincoln NB. Cognitive rehabilitation for spatial neglect following stroke. *Cochrane Database Syst Rev*. 2013:CD003586

12. Doron N, Rand D. Is unilateral spatial neglect associated with motor recovery of the affected upper extremity poststroke? A systematic review. *Neurorehabil Neural Repair*. 2019;33:179-187

13. Prvu Bettger J, Liu C, Gandhi DBC, Sylaja PN, Jayaram N, Pandian JD. Emerging areas of stroke rehabilitation research in low- and middle-income countries: A scoping review. *Stroke*. 2019;50:3307-3313

14. McDonald MW, Black SE, Copland DA, Corbett D, Dijkhuizen RM, Farr TD, Jeffers MS, Kalaria RN, Karayanidis F, Leff AP, Nithianantharajah J, Pendlebury S, Quinn TJ, Clarkson AN, O'Sullivan MJ. Cognition in stroke rehabilitation and recovery research: Consensus-based core recommendations from the second stroke recovery and rehabilitation roundtable. *Neurorehabil Neural Repair*. 2019;33:943-950

15. Pollock A, St George B, Fenton M, Firkins L. Top 10 research priorities relating to life after stroke--consensus from stroke survivors, caregivers, and health professionals. *Int J Stroke*. 2014;9:313-320

16. <http://dghs.gov.in/content/1363_3_NationalProgrammePreventionControl.aspx>.

17. <http://ncdirindia.org/stroke/BS_About.aspx#NSRP>.

18. Williamson JB, Lamb DG, Burtis DB, Haque S, E MZ, Kesayan T, Harciarek M, Heilman KM. Right hemispatial ipsilesional neglect with chronic right hemisphere strokes. *J Clin Exp Neuropsychol*. 2018;40:347-356

19. Ten Brink AF, Verwer JH, Biesbroek JM, Visser-Meily JMA, Nijboer TCW. Differences between left- and right-sided neglect revisited: A large cohort study across multiple domains. *J Clin Exp Neuropsychol*. 2017;39:707-723

20. Ogourtsova T, Archambault PS, Lamontagne A. Post-stroke visual neglect affects goal-directed locomotion in different perceptuo-cognitive conditions and on a wide visual spectrum. *Restor Neurol Neurosci*. 2018;36:313-331

21. van Nes IJ, van der Linden S, Hendricks HT, van Kuijk AA, Rulkens M, Verhagen WI, Geurts AC. Is visuospatial hemineglect really a determinant of postural control following stroke? An acute-phase study. *Neurorehabil Neural Repair*. 2009;23:609-614

22. Morone G, Matamala-Gomez M, Sanchez-Vives MV, Paolucci S, Iosa M. Watch your step! Who can recover stair climbing independence after stroke? *Eur J Phys Rehabil Med*. 2018;54:811-818

23. Zebhauser PT, Vernet M, Unterburger E, Brem AK. Visuospatial neglect - a theory-informed overview of current and emerging strategies and a systematic review on the therapeutic use of non-invasive brain stimulation. *Neuropsychol Rev*. 2019;29:397-420

24. Arya KN, Pandian S, Vikas, Puri V. Mirror illusion for sensori-motor training in stroke: A randomized controlled trial. *J Stroke Cerebrovasc Dis*. 2018;27:3236-3246

25. Arya KN. Underlying neural mechanisms of mirror therapy: Implications for motor rehabilitation in stroke. *Neurol India*. 2016;64:38-44

26. Tosi G, Romano D, Maravita A. Mirror box training in hemiplegic stroke patients affects body representation. *Front Hum Neurosci*. 2017;11:617

27. Pandian JD, Arora R, Kaur P, Sharma D, Vishwambaran DK, Arima H. Mirror therapy in unilateral neglect after stroke (must trial): A randomized controlled trial. *Neurology*. 2014;83:1012-1017

28. Thieme H, Bayn M, Wurg M, Zange C, Pohl M, Behrens J. Mirror therapy for patients with severe arm paresis after stroke--a randomized controlled trial. *Clin Rehabil*. 2013;27:314-324

29. Dohle C, Pullen J, Nakaten A, Kust J, Rietz C, Karbe H. Mirror therapy promotes recovery from severe hemiparesis: A randomized controlled trial. *Neurorehabil Neural Repair*. 2009;23:209-217

30. Ramachandran VS, Altschuler EL, Stone L, Al-Aboudi M, Schwartz E, Siva N. Can mirrors alleviate visual hemineglect? *Med Hypotheses*. 1999;52:303-305

31. Ambron E, Schettino LF, Coyle M, Jax S, Coslett HB. When perception trips action! The increase in the perceived size of both hand and target matters in reaching and grasping movements. *Acta Psychol (Amst)*. 2017;180:160-168

32. Ambron E, Jax S, Schettino LF, Coslett HB. Magnifying vision improves motor performance in individuals with stroke. *Neuropsychologia*. 2018;119:373-381

33. Winters C, van Wegen EE, Daffertshofer A, Kwakkel G. Generalizability of the maximum proportional recovery rule to visuospatial neglect early poststroke. *Neurorehabil Neural Repair*. 2017;31:334-342

34. Arya KN, Pandian S, Kumar D, Puri V. Task-based mirror therapy augmenting motor recovery in poststroke hemiparesis: A randomized controlled trial. *J Stroke Cerebrovasc Dis*. 2015;24:1738-1748

35. Arya KN, Pandian S, Kumar V. Effect of activity-based mirror therapy on lower limb motor-recovery and gait in stroke: A randomised controlled trial. *Neuropsychol Rehabil*. 2019;29:1193-1210

36. Ambron E, Jax S, Schettino L, Coslett HB. Increasing perceived hand size improves motor performance in individuals with stroke: A home-based training study. *PeerJ*. 2019;7:e7114

37. Sobrinho KRF, Santini ACM, Marques CLS, Gabriel MG, Neto EM, de Souza L, Bazan R, Luvizutto GJ. Impact of unilateral spatial neglect on chronic patient's post-stroke quality of life. *Somatosens Mot Res*. 2018;35:199-203

38. Fugl-Meyer AR, Jaasko L, Leyman I, Olsson S, Steglind S. The post-stroke hemiplegic patient. 1. A method for evaluation of physical performance. *Scand J Rehabil Med*. 1975;7:13-31

39. Tombaugh TN, McIntyre NJ. The mini-mental state examination: A comprehensive review. *J Am Geriatr Soc*. 1992;40:922-935

40. Lerdal A, Kottorp A, Gay CL, Grov EK, Lee KA. Rasch analysis of the beck depression inventory-ii in stroke survivors: A cross-sectional study. *J Affect Disord*. 2014;158:48-52

41. Richter P, Werner J, Heerlein A, Kraus A, Sauer H. On the validity of the beck depression inventory. A review. *Psychopathology*. 1998;31:160-168

42. Randomski MV, Catherine ATL. Occupational therapy for physical dysfunction. . 2008

43. O'Sullivan S, Schimitz TJ, Fulk GD. Physical rehabilitation (sixth edition). 2013

44. Duncan PW, Propst M, Nelson SG. Reliability of the fugl-meyer assessment of sensorimotor recovery following cerebrovascular accident. *Phys Ther*. 1983;63:1606-1610

45. Gladstone DJ, Danells CJ, Black SE. The fugl-meyer assessment of motor recovery after stroke: A critical review of its measurement properties. *Neurorehabil Neural Repair*. 2002;16:232-240

46. Sanford J, Moreland J, Swanson LR, Stratford PW, Gowland C. Reliability of the fugl-meyer assessment for testing motor performance in patients following stroke. *Phys Ther*. 1993;73:447-454

47. Chen P, Chen CC, Hreha K, Goedert KM, Barrett AM. Kessler foundation neglect assessment process uniquely measures spatial neglect during activities of daily living. *Arch Phys Med Rehabil*. 2015;96:869-876 e861

48. Chen P, Hreha K, Fortis P, Goedert KM, Barrett AM. Functional assessment of spatial neglect: A review of the catherine bergego scale and an introduction of the kessler foundation neglect assessment process. *Top Stroke Rehabil*. 2012;19:423-435

49. Downs S, Marquez J, Chiarelli P. The berg balance scale has high intra- and inter-rater reliability but absolute reliability varies across the scale: A systematic review. *J Physiother*. 2013;59:93-99

50. Blum L, Korner-Bitensky N. Usefulness of the berg balance scale in stroke rehabilitation: A systematic review. *Phys Ther*. 2008;88:559-566

51. Kollen B, Kwakkel G, Lindeman E. Time dependency of walking classification in stroke. *Phys Ther*. 2006;86:618-625

52. Mehrholz J, Wagner K, Rutte K, Meissner D, Pohl M. Predictive validity and responsiveness of the functional ambulation category in hemiparetic patients after stroke. *Arch Phys Med Rehabil*. 2007;88:1314-1319

53. Kwakkel G, Lannin NA, Borschmann K, English C, Ali M, Churilov L, Saposnik G, Winstein C, van Wegen EE, Wolf SL, Krakauer JW, Bernhardt J. Standardized measurement of sensorimotor recovery in stroke trials: Consensus-based core recommendations from the stroke recovery and rehabilitation roundtable. *Int J Stroke*. 2017;12:451-461

54. Banks JL, Marotta CA. Outcomes validity and reliability of the modified rankin scale: Implications for stroke clinical trials: A literature review and synthesis. *Stroke*. 2007;38:1091-1096

55. Chan AW, Tetzlaff JM, Altman DG, Laupacis A, Gotzsche PC, Krleza-Jeric K, Hrobjartsson A, Mann H, Dickersin K, Berlin JA, Dore CJ, Parulekar WR, Summerskill WS, Groves T, Schulz KF, Sox HC, Rockhold FW, Rennie D, Moher D. Spirit 2013 statement: Defining standard protocol items for clinical trials. *Ann Intern Med*. 2013;158:200-207

56. Boutron I, Altman DG, Moher D, Schulz KF, Ravaud P. Consort statement for randomized trials of nonpharmacologic treatments: A 2017 update and a consort extension for nonpharmacologic trial abstracts. *Ann Intern Med*. 2017;167:40-47
